# ANGPTL8 links refeeding to monocyte dynamics and metabolic inflammation via the CCL5-CCR5 axis

**DOI:** 10.1172/jci.insight.196605

**Published:** 2025-11-25

**Authors:** Ran-Ran Kan, Si-Yi Wang, Xiao-Yu Meng, Li Huang, Yu-Xi Xiang, Bei-Bei Mao, Hua-Jie Zou, Ya-Ming Guo, Li-Meng Pan, Pei-Qiong Luo, Yan Yang, Zhe-Long Liu, De-Lin Ma, Wen-Jun Li, Yong Chen, Dan-Pei Li, Xue-Feng Yu

**Affiliations:** 1 Division of Endocrinology, Department of Internal Medicine, Tongji Hospital, Tongji Medical College, Huazhong University of Science and Technology, Wuhan, China.; 2 Hubei Clinical Medical Research Center for Endocrinology and Metabolic Diseases, Hubei, China.; 3 Branch of National Clinical Research Center for Metabolic Diseases, Hubei, China.; 4 Computer Center, Tongji Hospital, Tongji Medical College, Huazhong University of Science and Technology, Wuhan, China.

**Keywords:** Immunology, Metabolism, Cell migration/adhesion, Chemokines, Monocytes

## Abstract

Metabolic inflammation is closely linked to dynamic changes in circulating monocyte populations, yet how nutritional signals regulate this process remains unclear. ANGPTL8, a hepatokine rapidly induced by refeeding, emerged as a key regulator of postprandial monocyte dynamics. We examined ANGPTL8 expression in human and murine fasting-refeeding models and manipulated ANGPTL8 expression specifically in hepatocytes to assess its role in metabolic inflammation and insulin resistance in obese mice. ANGPTL8 overexpression elevated levels of circulating monocytes and proinflammatory cytokines, while its deletion reduced these parameters and conferred metabolic benefits. Mechanistically, recombinant ANGPTL8 stimulated CCL5 production in bone marrow–derived macrophages via P38 signaling activation, promoting monocyte recruitment and proinflammatory macrophage polarization. These effects were mitigated by CCR5 antagonism. Rescue experiments demonstrated that CCL5 supplementation in *Angptl8*-deficient mice restored monocyte levels and inflammatory responses. Functionally, ANGPTL8 worsened insulin resistance and glucose intolerance in obese mice, effects that were reversed by its deletion and recapitulated by CCL5 administration. These findings suggest that ANGPTL8 functions as a nutritional checkpoint that links feeding status to monocyte-mediated inflammation through the CCL5-CCR5 axis. By driving monocytosis and proinflammatory macrophage activation, ANGPTL8 exacerbates metabolic dysfunction. Targeting the ANGPTL8-CCL5-CCR5 pathway may therefore offer a promising therapeutic strategy for managing obesity-related metabolic diseases.

## Abstract 2

ANGPTL8 links nutritional status to metabolic inflammation by driving monocyte recruitment through the CCL5–CCR5 axis.

## Introduction

Dietary intake is closely associated with low-grade chronic inflammation, a key contributor to various metabolic diseases, including type 2 diabetes, atherosclerosis, and hepatic steatosis (1). However, the molecular mechanisms underlying dietary modulation of systemic inflammation remain incompletely understood. Recent studies revealed that fasting-refeeding affects immune cell dynamics (2, 3). Dietary intake has been confirmed to be involved in regulating the size and composition of the circulating inflammatory monocyte pool in humans and animals (4). Fasting drastically reduces monocyte levels in circulation and peripheral tissues. In contrast, refeeding induces a marked increase in monocyte levels. However, investigations into mechanisms linking diet intake with monocyte regulation are scarce.

Angiopoietin-like protein 8 (ANGPTL8) is a protein that is secreted mainly from the liver and associated with lipid metabolism (5). Initial reports of ANGPTL8 described it as refeeding induced in fat and liver (RIFL), named for its dramatically increased expression after refeeding (6). While it is established that ANGPTL8 increases postprandial blood lipids, its other pathophysiological functions under refeeding states remain unclear. Previous studies revealed that other members of the ANGPTL family (ANGPTL2 and ANGPTL4) associated with monocyte migration and macrophage polarization (7). Our previous findings also identified that hepatocyte-specific *Angptl8* knockout reduces macrophage infiltration and resolves lipid accumulation and fibrosis progression in the livers of mice with nonalcoholic steatohepatitis (8). We also revealed the role of ANGPTL8 in the pathogenesis of diabetes-associated cognitive dysfunction and identified ANGPTL8 as a potential target for the management of this condition (9). We therefore sought to determine whether ANGPTL8 is a critical regulator of diet-induced inflammation and whether the effect of ANGPTL8 depends on its regulation of fasting/refeeding-associated immune cell dynamics.

In obesity, peripheral tissues are dysregulated by the infiltration of immune cells, associated with increased production of cytokines and chemokines. Particularly, proinflammatory monocyte-derived macrophage (MDM) accumulation is an important feature of obesity, correlating with local expression of inflammatory markers (10). Now, there is overwhelming evidence that inflammatory cytokines are directly associated with insulin resistance (1). Inhibition of monocyte infiltration to decrease the production of cytokines can disrupt the link between obesity and insulin resistance (1). Therefore, the mechanisms by which dietary intake increases the monocyte pool would be potential therapeutic targets for improve metabolic inflammation and insulin resistance.

Building on this foundation, we demonstrate here that ANGPTL8 functions as a central regulator of monocyte dynamics and a key promoter of metabolic inflammation and insulin resistance through facilitation of production of CCL5 (also known as regulated on activation, normal T cell expressed and secreted [RANTES]). Notably, the inhibition of ANGPTL8 significantly reduced monocyte levels at the refeeding state and effectively relieved the progression of obesity-related metabolic inflammation and insulin resistance in mice.

## Results

### Fasting-refeeding alters ANGPTL8 and monocyte levels in healthy humans and mice.

**Figure 1:**
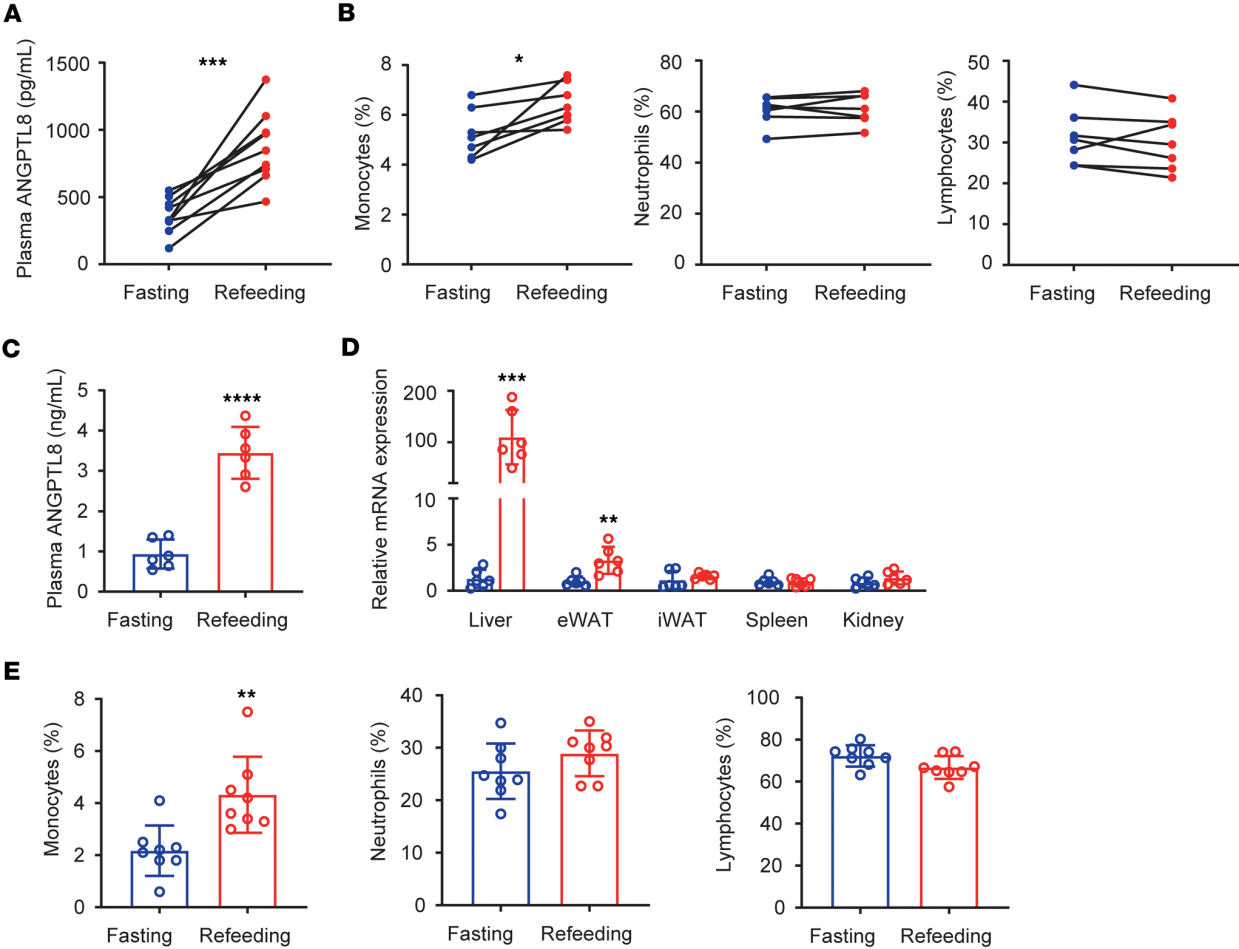
Nutritional state regulates ANGPTL8 and monocytes. (**A**) Plasma ANGPTL8 levels and (**B**) peripheral blood leukocyte populations of healthy humans in fasting-refeeding models (*n* = 9 individuals). (**C**) Plasma ANGPTL8 levels of mice in fasting-refeeding models (*n* = 6 mice/group). (**D**) *Angptl8* mRNA expression in different tissues (*n* = 6 mice/group). (**E**) Peripheral blood leukocyte populations of mice in fasting-refeeding models (*n* = 8 mice/group). All fasting/refeeding experiments were performed using a 12-hour fast during the dark phase (ZT2–14) followed by a 2-hour refeed. All samples are biologically independent replicates and n indicates the number of biologically independent samples examined. The data are shown as the mean ± SEM and were statistically analyzed by paired *t* test (**A** and **B**) or 2-tailed Student’s *t* test (**C**–**E**). All the *P* values were 2 sided. *P < 0.05, **P < 0.01, ***P < 0.001, ****P < 0.0001. iWAT, inguinal white adipose tissue; eWAT, epididymal white adipose tissue.

To explore whether ANGPTL8 was associated with changes in peripheral blood leukocyte populations, we tested plasma ANGPTL8 levels and profiled the composition of blood circulating immune cells of 9 healthy weight volunteers (mean age, 31 ± 7 years; BMI, 22.9 ± 2.1 kg/m^2^) after 20 hours of fasting (fasting state) and 2–4 hours after food intake (refeeding state) using automated blood cell counters. To control for circadian variations and dietary intake, all blood samples were drawn at the same time of the day (12:00 am), and volunteers were given standardized food. Strikingly, we found that refeeding led to a significant increase in plasma ANGPTL8 (from 362.93 ± 133.61 pg/mL to 872.58 ± 269.59 pg/mL) and circulating monocytes (from 5.24% ± 0.99% to 6.47% ± 0.83%) compared with the fasting state, whereas neutrophil and lymphocyte counts were not significantly altered (Figure 1, A and B).

We then asked whether similar dynamics also occurred in mice. We chose a 2-hour refeeding protocol after 12 hours of fasting during the dark period (Zeitgeber 2–14 [ZT2–14]), which is comparable to overnight fasting for humans and is less stressful fasting strategy in animals (11). We found that both plasma levels (from 0.94 ± 0.36 ng/mL to 3.45 ± 0.65 ng/mL) and hepatic mRNA expression of ANGPTL8 were significantly elevated upon refeeding (Figure 1, C and D). Importantly, refeeding also led to significant increase of circulating monocytes (from 2.18% ± 0.97% to 4.33% ± 1.46%) compared with fasting state, whereas neutrophils and lymphocytes were not significantly affected (Figure 1E).

### ANGPTL8 overexpression increases monocytes and inflammatory cytokines in fasting mice.

**Figure 2:**
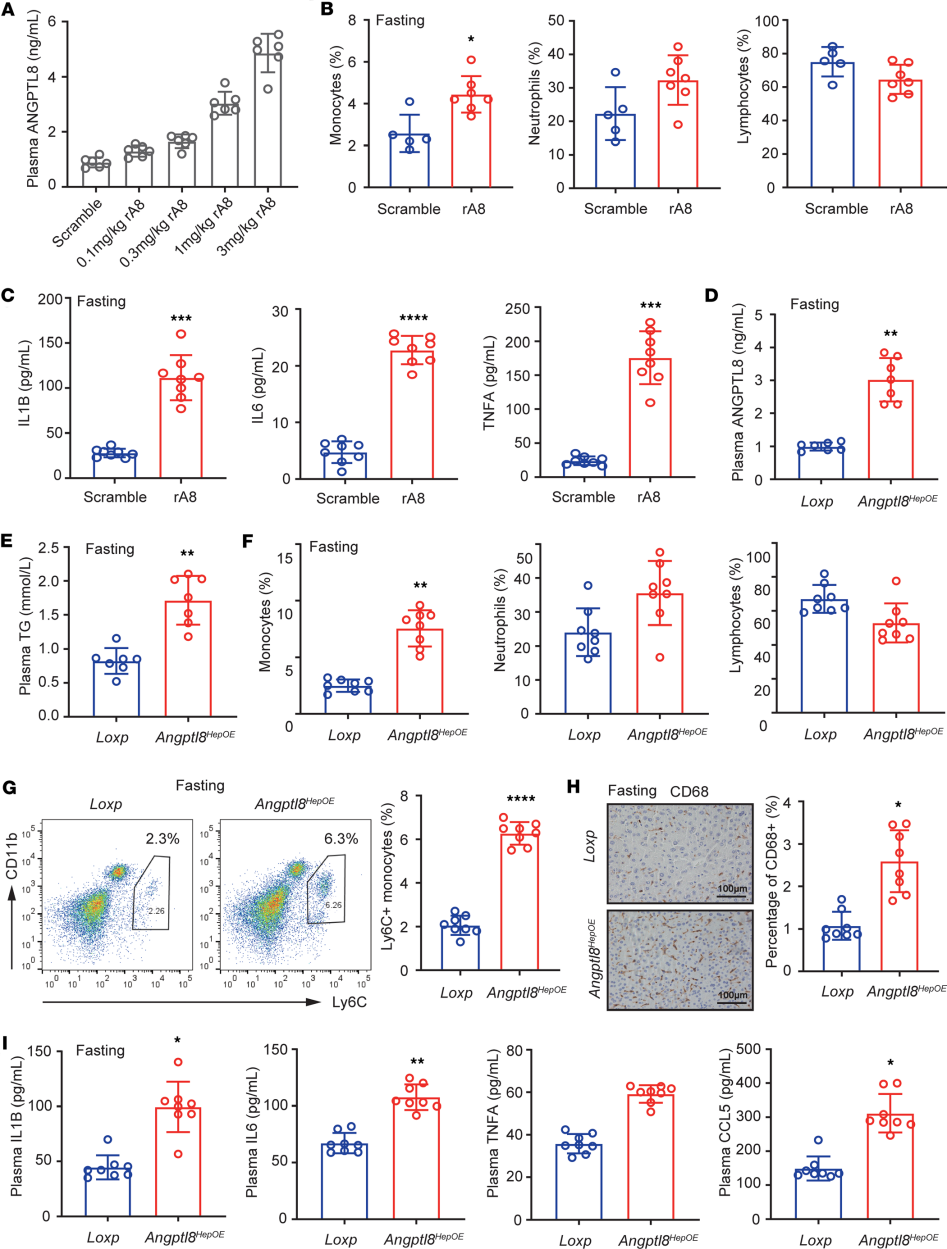
ANGPTL8 overexpression elevates monocytes and inflammation. (**A**) Plasma ANGPTL8 levels of fasting mice with i.p. injections of rA8 at different doses (0.1, 0.3, 1.0, 3.0 mg/kg) (*n* = 6 mice/group). (**B**) Peripheral blood leukocyte populations and (**C**) plasma chemokines levels of fasting mice with rA8 injection (*n* = 8 mice/group). (**D**) Plasma ANGPTL8, (**E**) TG levels, and (**F**) peripheral blood leukocyte populations of fasting *Angptl8^HepOE^* mice (*n* = 8 mice/group). (**G**) Flow cytometry analysis of proinflammatory Ly6C^+^ monocytes in livers of fasting *Loxp* and *Angptl8^HepOE^* mice. (**H**) Representative liver images of liver sections and quantification of CD68 staining of fasting *Loxp* and *Angptl8^HepOE^* mice (*n* = 8 mice/group). Scale bar: 100 μm. (**I**) Plasma chemokines levels of fasting *Loxp* and *Angptl8^HepOE^* mice (*n* = 8 mice/group). Data are shown as the mean ± SEM and were statistically analyzed by 2-tailed Student’s *t* test (**B**–**F**, **H**, and **I**). All samples are biologically independent replicates, and *n* indicates the number of biologically independent samples examined. Data shown are representative of 3 independent experiments with similar results (**G**). All the *P* values were 2 sided. *P < 0.05, **P < 0.01, ***P < 0.001, ****P < 0.0001. rA8, recombinant ANGPTL8.

To elucidate whether ANGPTL8 is involved in the regulation of diet-induced immune cell dynamics in vivo, we i.p. injected 12-hour-fasting male C57BL/6J mice with varying concentrations of recombinant ANGPTL8 protein (rA8). Blood was collected 2 hours after injection for analysis. A single injection of rA8 (1.0 mg/kg) increased plasma ANGPTL8 levels to 3.0–4.0 ng/mL (Figure 2A), a range comparable to the incremental change observed in refed mice (Figure 1C). We found that treatment of fasting mice with 1 mg/kg rA8 induced a significant increase in circulating monocytes (Figure 2B) and inflammatory cytokines (Figure 2C). Neutrophil and lymphocyte levels did not show statistically significant changes, although there was a trend toward increased neutrophils and decreased lymphocytes (Figure 2B). Based on the concept that nutrient availability affects bone marrow precursor metabolism (4), we explored whether ANGPTL8 influences myelopoiesis by altering fuel delivery or usage. Our results indicated that triglyceride and glucose levels in the bone marrow reflected systemic levels following rA8 administration, and the nutrient uptake capacity of bone marrow CXCR4^+^ monocytic precursors remained unchanged (Supplemental Figure 1; supplemental material available online with this article; https://doi.org/10.1172/jci.insight.196605DS1).

Considering that the refeeding-induced increase of ANGPTL8 was mainly from liver (Figure 1D), to examine the physiological effects of endogenous ANGPTL8 on immune cell dynamics, we generated mice with hepatocyte-specific overexpression of ANGPTL8 (hereafter referred to as *Angptl8^HepOE^* mice). Compared with their *Loxp* littermates, *Angptl8^HepOE^* mice exhibited 3-fold increase in plasma ANGPTL8 and TG levels at fasting state (Figure 2, D and E); meanwhile, circulating monocytes were increased, but neutrophils and lymphocytes were not significant changed (Figure 2F). Importantly, fasting *Angptl8^HepOE^* mice also exhibited a significant increase of proinflammatory Ly6C^+^ monocytes and in peripheral tissues such as livers (Figure 2G). Based on previous studies, circulating monocytes have been suggested to give rise to tissue infiltrating macrophages (12). As expected, we found that the livers of *Angptl8^HepOE^* mice harbored more macrophages than their *Loxp* littermates (Figure 2H). In addition, we found that the plasma levels of inflammatory cytokines and chemokines were significantly increased in *Angptl8^HepOE^* mice comparing with those of *Loxp* littermates, including IL1B, IL6, TNFA, and CCL5 (Figure 2I).

To assess if monocytosis and inflammation were due to ANGPTL8-induced hypertriglyceridemia, we treated *Angptl8^HepOE^* mice and controls with fenofibrate. Although fenofibrate normalized triglyceride levels, it did not reduce circulating monocytes or proinflammatory cytokines, indicating that ANGPTL8’s proinflammatory effects are independent of its effect on triglycerides (Supplemental Figure 2). To investigate if the immunomodulatory pathway depends on the ANGPTL3/8 complex, we studied ANGPTL8 effects in *Angptl3*-knockdown mice. Removing ANGPTL3 eliminated ANGPTL8’s hypertriglyceridemic effect but did not affect its ability to cause monocytosis, activate P38, or increase inflammatory cytokines (Supplemental Figure 4). These results suggested that ANGPTL8 independently mediated the fasting/refeeding-induced dynamics of monocyte and cytokine production. Restoring plasma ANGPTL8 levels by administration of recombinant protein or genetic overexpression rescued decreasing monocyte numbers in fasting mice.

### ANGPTL8 promotes monocyte migration via the CCL5-CCR5 axis.

**Figure 3:**
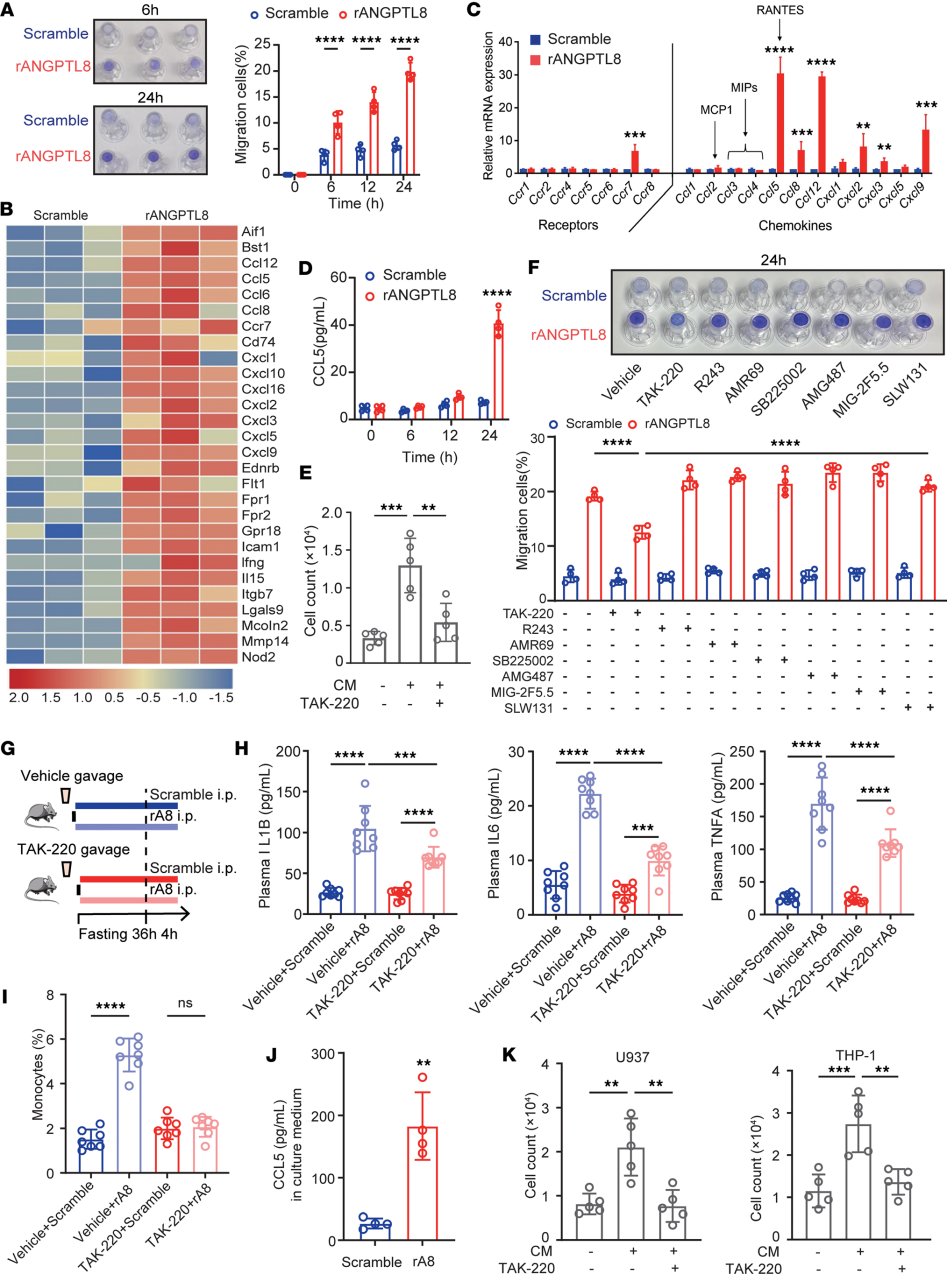
ANGPTL8 promotes monocyte migration via CCL5-CCR5. (**A**) Representative images and quantification of monocyte migration induced by conditioned medium (CM) from BMDMs pretreated with or without rA8 over time (*n* = 4). (**B**) Heatmap of chemokine-related DEGs (adjusted *P* < 0.05) in BMDMs cultured with or without rA8 (40 nM) stimulation for 24 hours (*n* = 3). (**C**) mRNA expression of chemokines and their receptors after BMDMs were treated with rA8 or scrambled protein (*n* = 6 cells examined over 3 independent experiments). (**D**) CCL5 levels in CM from BMDMs with rA8 stimulation over time (*n* = 4). (**E**) Migration of BMDMs with or without CCR5 antagonist (TAK-220) before rA8 stimulation (*n* = 5 cells examined over 3 independent experiments). (**F**) Effects of chemokine antagonists on migration of BMDMs with rA8 treatment (*n* = 4). (**G**) Experimental scheme for CCR5 antagonist (TAK-220) gavage and rA8 injection in *Loxp* and *Angptl8^HepOE^* mice. (**H**) Plasma proinflammation cytokine levels and (**I**) circulating monocyte populations of indicated groups (*n* = 8 mice/group). (**J**) CCL5 levels in CM from human MDMs with or without rA8 stimulation (*n* = 4). (**K**) Migration of U937 and THP-1 cells pretreated with or without CCR5 antagonist (TAK-220) before rA8 stimulation (*n* = 5 cells examined over 3 independent experiments). Migrated cells were fixed and stained with 0.1% crystal violet (**A** and **F**). The data are shown as the mean ± SEM and were statistically analyzed by 2-tailed Student’s *t* test (**A**, **C**, **D**, **F**, and **J**) or 1-way ANOVA with Tukey’s multiple-comparison test (**E**, **H**, **I**, and **K**). All samples are biologically independent replicates, and *n* indicates the number of biologically independent samples examined. All the *P* values were 2 sided, and adjustments were made for multiple comparisons. *P < 0.05, **P < 0.01, ***P < 0.001, ****P < 0.0001. DEGs, differentially expressed genes; MDMs, monocyte-derived macrophages; rA8, recombinant ANGPTL8; MCP1, monocyte chemoattractant protein-1 (CCL2); MIPs, macrophage inflammatory proteins, RANTES, regulated on activation normal T cell expressed and secreted (CCL5); CM, conditioned medium; TAK-220, CCR5 antagonist; R243, CCR8 antagonist; AMR69, CCL12 inhibitor; SB225002, CXCR2 antagonist; AMG487, CXCR3 antagonist; MIG-2F5.5, anti-CXCL9 antibody; SLW131, CCR7 antagonist.

Previously published data suggested that dietary intake affects the circulating inflammatory monocyte pool through regulation of CCL5 production (13). Considering that *Angptl8^HepOE^* mice exhibited higher fasting CCL5 levels than *Loxp* controls (Figure 2I), we asked whether ANGPTL8 regulated postprandial circulating monocytes by increasing CCL5 secretion. Conditioned media (CM) collected from rA8-treated bone marrow–derived macrophages (hereafter referred to as BMDMs) induced significantly greater monocyte migration, with the most pronounced effect observed at 24 hours (Figure 3A). Based on this, subsequent migration assays were performed following 24-hour rA8 treatment. It has been accepted that macrophages are the predominant source of CCL5 (14, 15); we further analyzed ANGPTL8-induced changes in the transcriptional profile of BMDMs through mRNA sequencing to identify the potential functions of ANGPTL8. We thus treated BMDMs with rA8 and found that ANGPTL8 upregulated mRNA expression of several inflammation-associated chemokines and their receptors (Figure 3B), with CCL5 showing the most pronounced increase (Figure 3C). Meanwhile, in the CM of rA8-treated BMDMs, CCL5 levels increased in a time-dependent manner, reaching a peak at 24 hours (Figure 3D). More importantly, it can be abrogated by CCR5 antagonist (TAK-220) (Figure 3E), suggesting that the migration-promoting effect of ANGPTL8 was dependent on the CCL5-CCR5 signaling axis. To determine whether other upregulated cytokines also contributed to monocyte migration, we tested antagonists for CCR8 (R243), CCL12 (AMR69), CXCR2 (SB225002), CXCR3 (AMG487), CXCL9 (MIG-2F5.5), and CCR7 (SLW131). None of these inhibitors affected 24-hour rA8-induced monocyte migration (Figure 3F), suggesting that the CCL5-CCR5 axis functions as an independent mediator of this effect. Likewise, we did not observe any effect of all the antagonists on monocyte migration induced by 6-hour rA8-treatment of BMDMs (Supplemental Figure 3).

The in vivo relevance of these findings was corroborated in mice with disrupted CCL5-CCR5 signaling (Figure 3, G–I). Pharmacological disruption of CCL5 signaling (TAK-220) shortly before rA8 injection reduced the rA8-induced increase in fasting proinflammatory cytokines (Figure 3H) and circulating monocytes (Figure 3I). To further verify these findings in humans, we isolated and differentiated primary human MDMs. Consistent with results in mice, CM collected from rA8-treated human MDMs, exhibiting significantly higher CCL5 levels (Figure 3J), evoked more U937 and THP-1 cell migration compared with CM from controls, which was also abrogated by CCR5 antagonist (TAK-220) (Figure 3K).

### ANGPTL8 activates P38 to drive CCL5 secretion and macrophage polarization.

**Figure 4:**
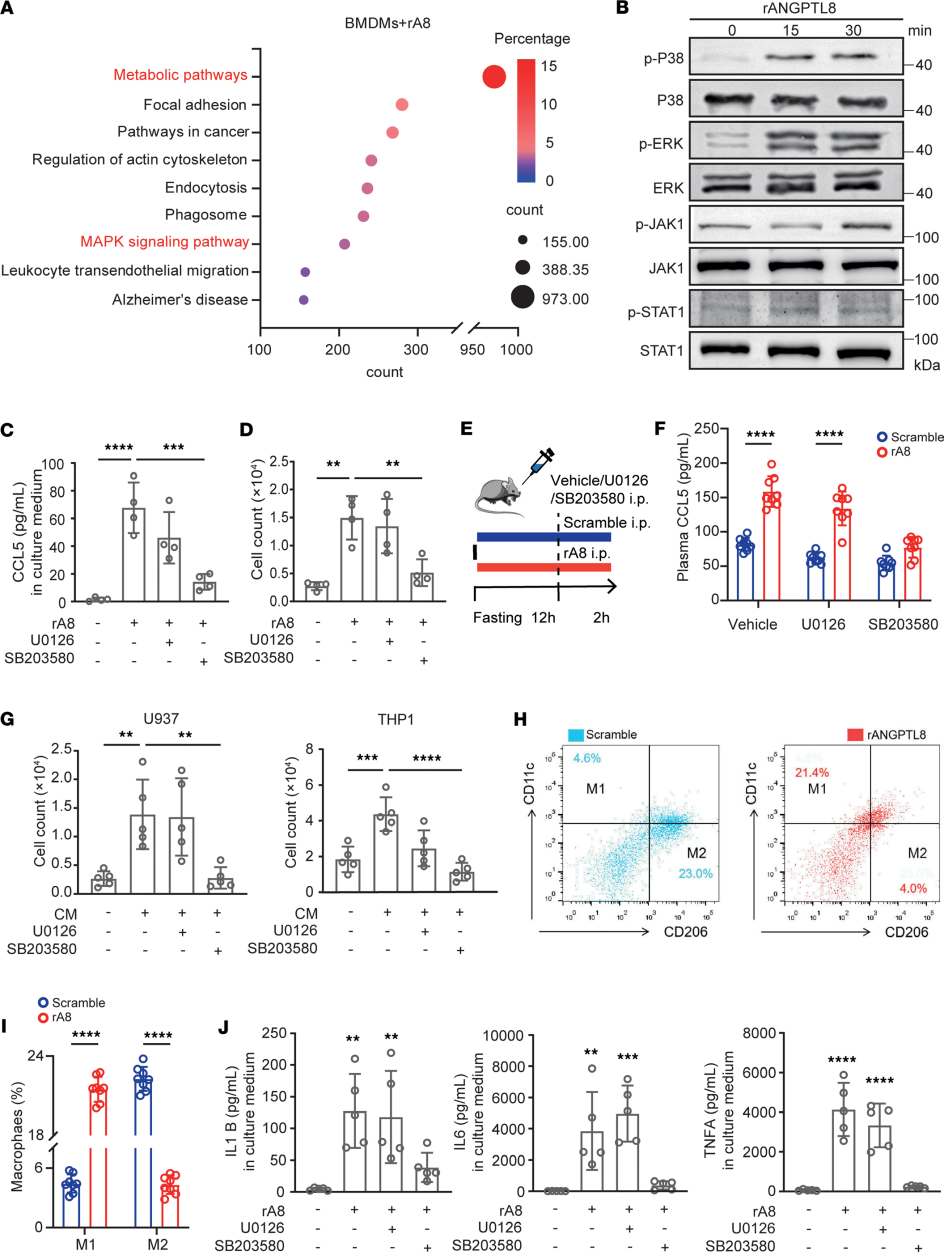
ANGPTL8 activates P38 to induce CCL5 and macrophage polarization. (**A**) Pathway enrichment analysis in BMDMs treated with rA8 (*n* = 3). (**B**) Western blot analysis of the phosphorylation of signaling molecules in BMDMs with rA8 stimulation. (**C**) CCL5 levels in the CM and (**D**) migration of BMDMs pretreated with phosphorylation inhibitors before rA8 stimulation (*n* = 6 cells examined over 3 independent experiments). U0126, p-ERK inhibitor; SB203580, p-P38 inhibitor. (**E**) Experimental scheme for phosphorylation inhibitor injection before rA8 stimulation in wild-type mice. (**F**) Plasma CCL5 levels of indicated groups (*n* = 8 mice/group). (**G**) Migration of U937 and THP-1 cells pretreated with or without phosphorylation inhibitors before rA8 stimulation (*n* = 5 cells examined over 3 independent experiments). (**H**) Flow cytometry analysis of M1 (proinflammation phenotype) macrophages (CD11b^hi^CD206^lo^) and M2 (antiinflammation phenotype) macrophages (CD11b^lo^CD206^hi^) in livers of wild-type mice with scramble or rA8 injection. (**I**) Quantification of the proportions of M1 and M2 macrophages. (**J**) Proinflammation chemokines levels in the CM from BMDMs pretreated with or without phosphorylation inhibitors before rA8 stimulation (*n* = 5 cells examined over 3 independent experiments). The data are shown as the mean ± SEM and were statistically analyzed by 1-way ANOVA with Tukey’s multiple-comparison test (**C**, **D**, **G**, and **J**) or 2-tailed Student’s *t* test (**F** and **I**). All samples are biologically independent replicates, and *n* indicates the number of biologically independent samples examined. Data shown are representative of 3 independent experiments with similar results (**B** and **H**). All the *P* values were 2 sided, and adjustments were made for multiple comparisons. **P < 0.01, ***P < 0.001, **** *P* < 0.0001. CM, conditioned medium; kDa, relative molecular weight in kilodaltons.

To figure out signals that could mediate the promoting effects of ANGPTL8 on CCL5 production, we tested the phosphorylation of downstream signals, which revealed the MAPK pathway to be markedly enriched (Figure 4A). Given that P38 and ERK are key components of the MAPK family, we next performed Western blot analysis to assess the phosphorylation levels of these 2 pathways, confirming their upregulation upon rA8 stimulation (Figure 4B). Furthermore, SB203580 (a P38 inhibitor) abrogated the rA8-induced CCL5 increase in culture medium of BMDMs (Figure 4C) and abolished the migration-promoting effect of CM on monocytes (Figure 4D), whereas U0126 (ERK inhibitor) failed to have obvious effect (Figure 4, C and D). Likewise, we found that inhibition of P38, rather than ERK signaling in vivo, reduced plasma CCL5 levels which evoked by rA8 injection (Figure 4, E and F). Therefore, ANGPTL8-induced CCL5 production depends on P38 signaling. Consistent with results in mice, CM collected from rA8-treated human MDMs evoked more U937 and THP-1 cell migration compared with CM from controls, which was also abrogated by P38 inhibitor (SB203580) (Figure 4G).

Intriguingly, while STAT1 phosphorylation remained unaffected in rA8-stimulated BMDMs (Figure 4B), flow cytometric analysis and its quantification revealed a significant increase in M1-polarized (inflammatory) macrophages, accompanied by a decrease in M2-polarized (antiinflammatory) macrophages (Figure 4, H and I). Furthermore, to evaluate the effect of ANGPTL8 on different macrophage subsets, we stimulated prepolarized M0, M1, and M2 BMDMs with rA8. Our results indicate that ANGPTL8 treatment further enhanced the expression of proinflammatory genes (including *Il1b*, *Il6*, and *Tnfa*) in M0 and M2 macrophages. In contrast, it had a negligible additional effect on the already activated M1 macrophages (Supplemental Figure 5). These results suggest that ANGPTL8 preferentially enhances inflammatory responses in nonclassically activated macrophages. Notably, the levels of proinflammatory cytokines (IL1B, IL6, and TNFA) in the conditioned medium from rA8-treated BMDMs were specifically reduced by the P38 inhibitor (SB203580), but not by the ERK inhibitor (U0126), indicating that M1 polarization is mediated through the P38 signaling pathway (Figure 4J).

### ANGPTL8 overexpression aggravates metabolic inflammation via CCL5-CCR5.

**Figure 5:**
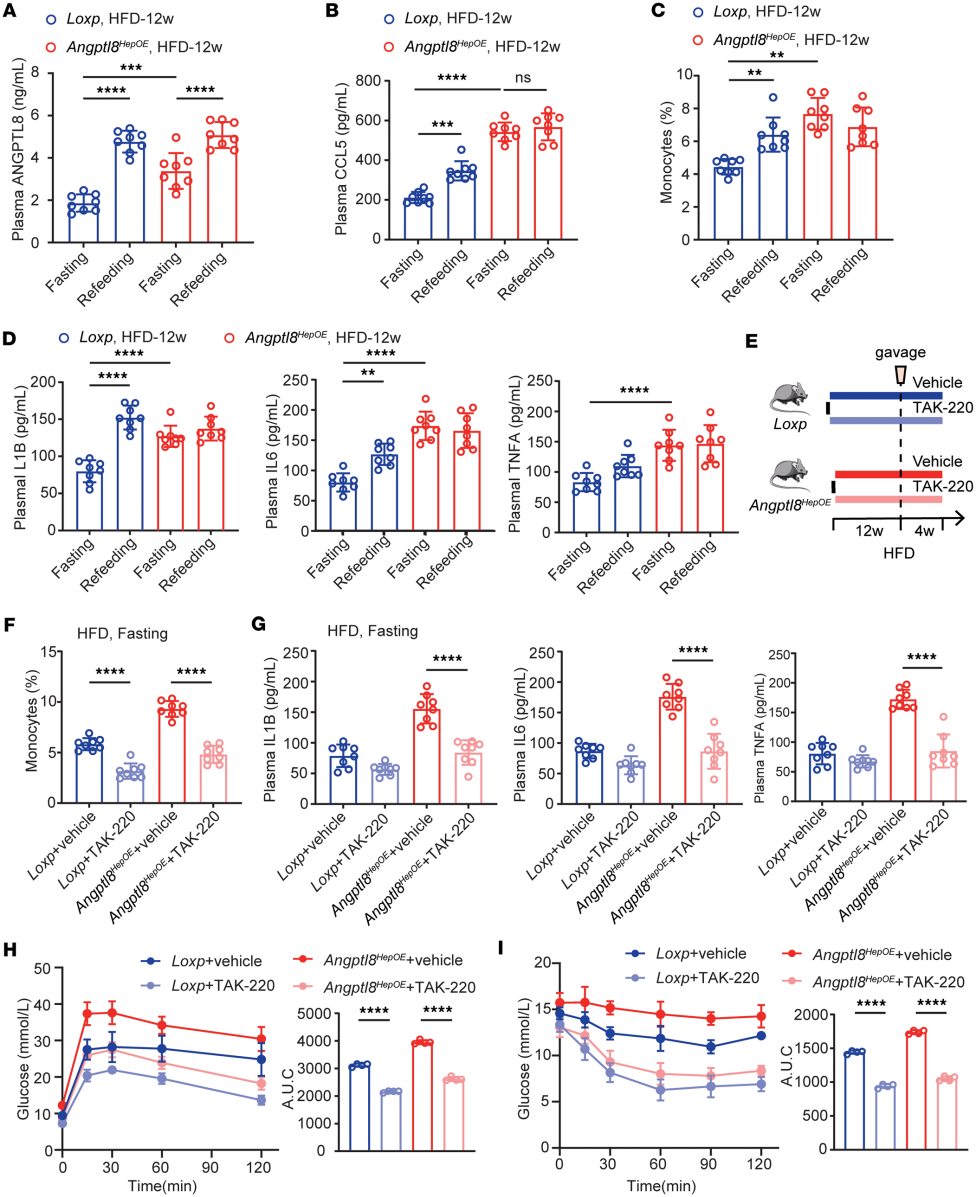
ANGPTL8 exacerbates metabolic inflammation via CCL5-CCR5. Effect of fasting-refeeding in the (**A**) plasma ANGPTL8 and (**B**) CCL5 levels of HFD-induced obese mice (*n* = 8 mice/group). (**C**) Circulating monocyte populations and (**D**) proinflammation cytokines of fasting-refeeding mice fed with HFD. (**E**) Experimental scheme for CCR5 antagonist (TAK-220) gavage in *Loxp* and *Angptl8^HepOE^* mice with fed with HFD. (**F**) Circulating monocyte populations and (**G**) proinflammation cytokine levels of the indicated groups (*n* = 8 mice/group). (**H**) IPGTTs and (**I**) ITTs of the indicated groups (*n* = 4 mice/group). All fasting/refeeding experiments were performed using a 12-hour fast during the dark phase (ZT2–14) followed by a 2-hour refeed. The data are shown as the mean ± SEM and were statistically analyzed by 1-way ANOVA with Tukey’s multiple-comparison test. All samples are biologically independent replicates, and *n* indicates the number of biologically independent samples examined. All the *P* values were 2 sided, and adjustments were made for multiple comparisons. **P < 0.01, ***P < 0.001, ****P < 0.0001. HFD, high-fat diet; TAK-220, CCR5 antagonist; IPGTTs, i.p. glucose tolerance tests; ITTs, insulin tolerance tests; AUC, area under curve.

To elucidate whether overexpression of ANGPTL8 aggravated obesity-related low-grade chronic inflammation and insulin resistance, *Angptl8^HepOE^* mice, together with their *Loxp* littermates (as controls), were challenged with a high-fat diet (HFD) for 12 weeks. Plasma ANGPTL8 and CCL5 levels as well as circulating monocytes were significantly increased in HFD-feeding *Angptl8^HepOE^* mice contrasted with controls, especially at fasting state (Figure 5, A–C). Meanwhile, fasting *Angptl8^HepOE^* mice exhibited more severe metabolic inflammation, determined by markedly higher fasting cytokine plasma levels (Figure 5D). The blunted response to refeeding in *Angptl8^HepOE^* mice suggests that the proinflammatory state in these animals is already maximally elevated under fasting conditions, leaving little capacity for further activation.

In light of the reported importance of the CCL5-CCR5 axis in metabolic inflammation (15, 16), we aimed at comparing the effect of CCR5 antagonist on HFD-feeding mice. Therefore, we designed a 4-week pharmacological treatment protocol on HFD-feeding *Loxp* and *Angptl8^HepOE^* mice (Figure 5E). Counts of fasting circulating monocytes were reduced by CCR5 antagonist delivery both in HFD-feeding *Loxp* and *Angptl8^HepOE^* mice (Figure 5F). Of note, while it was without significant effect on plasma IL1B and TNFA levels of 12-week-HFD *Loxp* mice, CCR5 antagonist was sufficient to lower plasma proinflammatory cytokines (IL1B, IL6, and TNFA) of *Angptl8^HepOE^* mice fed a HFD, suggesting that ANGPTL8-induced metabolic inflammation was dependent on the CCL5-CCR5 axis (Figure 5G).

The involvement of inflammation in insulin resistance has been shown previously (1). To validate our findings further, we performed i.p. glucose and insulin tolerance tests (IPGTTs and ITTs, respectively) and found that HFD-feeding *Angptl8^HepOE^* mice exhibited exacerbated insulin resistance relative to controls, as evident from higher blood glycemia detected throughout the time course of the experiment and lower glucose-lowering effect of insulin (Figure 5, H and I). Observations that CCR5 antagonist improves ANGPTL8-induced metabolic inflammation prompted us to further investigate whether it functions in resistance to associated insulin resistance. As expected, IPGTTs and ITTs revealed that CCR5 disruption protected *Angptl8^HepOE^* mice from serious insulin resistance due to HFD, suggesting that CCL5-CCR5 axis is necessary for obesity-mediated insulin resistance in *Angptl8^HepOE^* mice (Figure 5, H and I).

### Deleting ANGPTL8 alleviates HFD-induced inflammation and insulin resistance.

**Figure 6:**
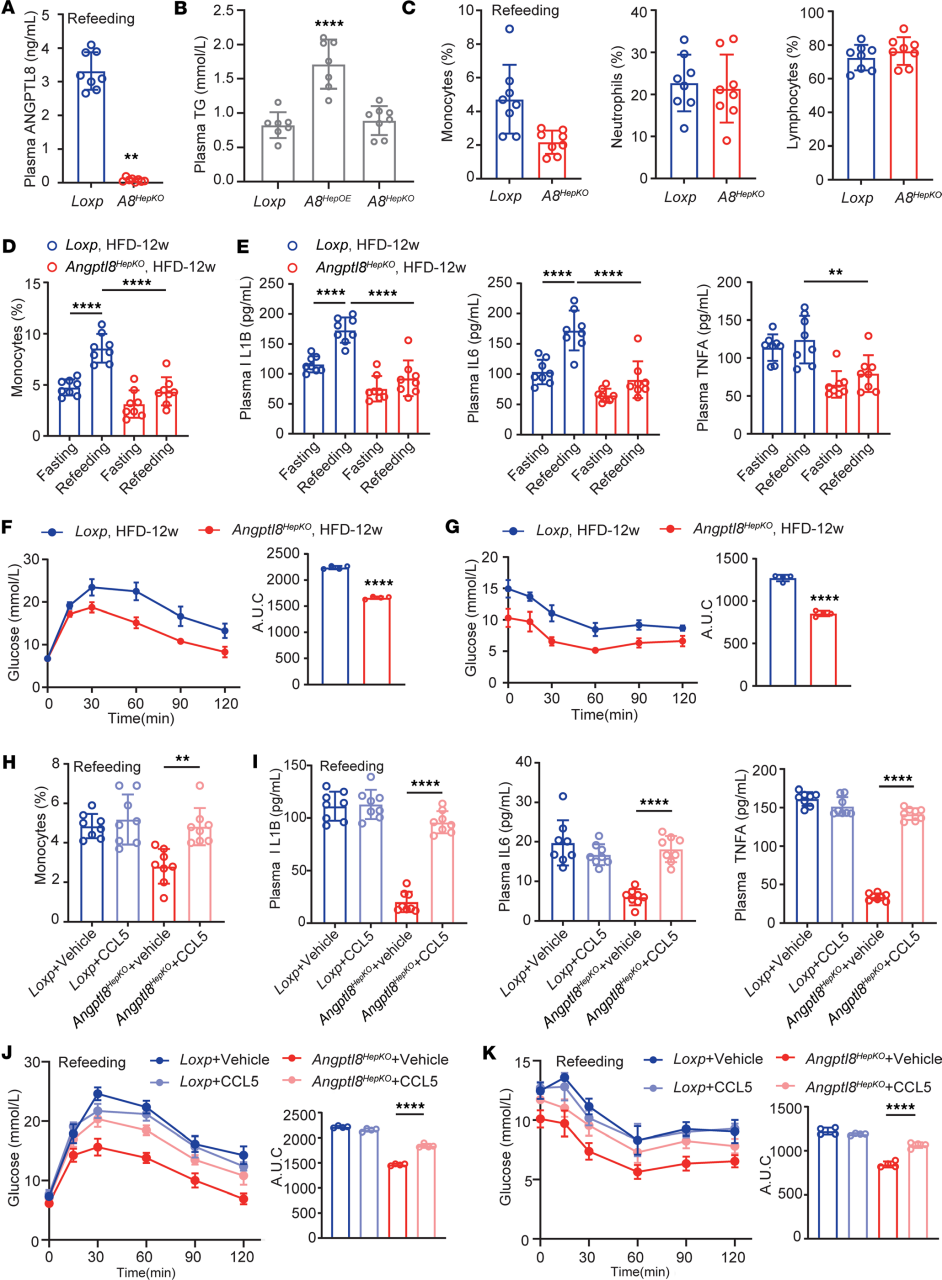
Deleting ANGPTL8 reduces inflammation and insulin resistance. (**A**) Circulating ANGPTL8 levels, (**B**) plasma TG levels, and (**C**) leukocyte populations of refeeding *Angptl8^HepKO^* and *Loxp* mice (*n* = 8 mice/group). Effect of fasting-refeeding in the (**D**) circulating monocyte populations and (**E**) proinflammation cytokine levels of *Angptl8^HepKO^* and *Loxp* mice fed with HFD (*n* = 8 mice/group). (**F**) IPGTTs and (**G**) ITTs of *Angptl8^HepKO^* and *Loxp* mice fed with HFD (*n* = 4 mice/group). (**H**) Circulating monocytes and (**I**) plasma cytokines of refeeding *Angptl8^HepKO^* and *Loxp* mice with or without CCL5 injection (*n* = 8 mice/group). (**J**) IPGTTs and (**K**) ITTs of refeeding *Angptl8^HepKO^* and *Loxp* mice with or without CCL5 injection (*n* = 4 mice/group). All fasting/refeeding experiments were performed using a 12-hour fast during the dark phase (ZT2–14) followed by a 2-hour refeed. The data are shown as the mean ± SEM and were statistically analyzed by 2-tailed Student’s *t* test (**A**, **C**, **F**, **G**) or 1-way ANOVA with Tukey’s multiple-comparison test (**B**–**E** and **H**–**K**). All samples are biologically independent replicates, and *n* indicates the number of biologically independent samples examined. All the *P* values were 2 sided, and adjustments were made for multiple comparisons. **P < 0.01, ****P < 0.0001. HFD, high-fat diet; IPGTTs, i.p. glucose tolerance tests; ITTs, insulin tolerance tests; AUC, area under curve.

Hepatocyte-specific *Angptl8*-knockout mice were generated to investigate whether deletion of ANGPTL8 reduced peripheral blood leukocyte populations. Refeeding *Angptl8^HepKO^* mice exhibited significantly decreased plasma ANGPTL8 levels, with no notable effect on plasma triglycerides (Figure 6, A and B) or gut-derived LPS (Supplemental Figure 6). Circulating monocytes were also markedly reduced in these mice, whereas neutrophil and lymphocyte populations remained unaffected (Figure 6C). However, we were unable to detect significant differences in plasma proinflammatory cytokine levels between normal diet–fed *Angptl8^HepKO^* mice and controls (Supplemental Figure 7). Importantly, plasma CCL5 protein levels were significantly induced by refeeding in *Loxp* mice, but this induction was completely abrogated in *Angptl8^HepKO^* mice (Supplemental Figure 8), demonstrating that ANGPTL8 is essential for the feeding-induced upregulation of CCL5.

To investigate the effect of *Angptl8* deletion on obesity-related metabolic inflammation and insulin resistance, we exposed *Angptl8^HepKO^* mice and their control (*Loxp*) littermates to a HFD for 12 weeks to induce metabolic inflammation. Notably, *Angptl8* deletion did not alter body weight gain in these mice (Supplemental Figure 9), indicating that the observed metabolic and immune phenotypes were independent of changes in overall adiposity. *Angptl8^HepKO^* mice showed impaired the fasting-refeeding dynamics monocytes, as evidenced by lower portion of refeeding monocytes (Figure 6D). Meanwhile, deletion of *Angptl8* conferred resistance to HFD-induced metabolic inflammation, as evidenced by significantly attenuated plasma levels of IL1B, IL6, and TNFA in *Angptl8^HepKO^* mice versus controls, regardless of nutritional status (Figure 6E). Additionally, IPGTTs and ITTs revealed that insulin resistance was markedly improved by *Angptl8* depletion (Figure 6, F and G). Administration of recombinant CCL5 at a determined effective dose (Supplemental Figure 8B) fully restored monocyte numbers and inflammatory cytokine levels in the knockout mice (Figure 6, H and I). Moreover, this CCL5 reconstitution abolished the improved insulin sensitivity in *Angptl8^HepKO^* mice, worsening their glucose tolerance and insulin resistance to the level of controls (Figure 6, J and K).

These data pointed to ANGPTL8 as a potential therapeutic target for efficient inhibition of postprandial circulating monocyte recruitment and obesity-related metabolic inflammation as well as insulin resistance.

## Discussion

Cytometry profiling of blood cells isolated from healthy humans and mice during fasting and refeeding revealed an association between dietary intake and the circulating monocytes. Here, we showed that ANGPTL8, a protein significantly increased after dietary intake, regulates the inflammatory activity and egress of monocytes to the blood circulation.

Egress of monocytes to the blood circulation is a critical step in the inflammatory cascade in peripheral tissues (17). Although it was previously thought that postprandial expansion of circulating monocytes under physiological conditions might exert protective effects — for instance, by efficiently phagocytosing lipid particles (18) and contributing to postprandial lipid clearance (19) — accumulating evidence suggests that the differentiation of monocytes into macrophages within the vascular intima, accompanied by the uptake of lipid particles, including oxidized lipoproteins, and transformation into lipid-laden foam cells, contributes to the pathogenesis of various metabolic disorders (20). Moreover, monocytes are important producers of proinflammatory cytokines and play a critical role in the induction and maintenance of inflammation. Therefore, it is conceivable that modulation of peripheral monocyte load in blood and tissues by reducing migration decreases susceptibility to pathological inflammatory disease. Our study reveals that ANGPTL8 is a critical modulator of postprandial monocyte pool and investigates its potential as a therapeutic target for nutrient-induced inflammatory diseases.

Obesity-related inflammation (metabolic inflammation) is considered instrumental in the development of insulin resistance (16), and it is a modifiable risk factor associated with the cardiometabolic disorders (1). Circulating monocytes are increased in overweight and obese humans, thus producing inflammatory cytokines and provoking chronic low-grade inflammation. We found that ANGPTL8 deletion reduces peripheral proinflammatory cells, leading to an overall improved inflammatory profile as well as insulin sensitivity in obese mice. This result was consistent with our previous clinical studies, which implied that serum ANGPTL8 levels are associated with an increased risk of all-cause mortality and could be used as a potential biomarker for the prediction of death in patients with diabetes (21). Removing part of the C-terminal coiled-coil domain of ANGPTL8 might disrupt the complex formation with ANGPTL3 and ANGPTL4, influencing lipid partitioning by altering the inhibitory effect on LPL (22). The tight association of ANGPTL8 with ANGPTL3 and ANGPTL4 has posed challenges in disentangling the independent effects of ANGPTL8 on metabolic dysregulation (23). Considering the association between inflammation and cardiovascular mortality, we thus speculated that reduction of ANGPTL8 would decrease the cardiovascular disease and its related mortality. This speculation needed more evidence to support.

In obese mice and humans, circulating CCL5 levels correlate positively with the degree of corpulence and insulin resistance (24, 25). CCL5- and CCR5-knockout mice show reduced adipose tissue macrophages, lower levels of inflammatory mediators, and improved insulin sensitivity (26, 27). Notably, the mechanism(s) triggering abnormally high CCL5 levels in obesity remains unclear. We propose a mechanism where the hepatokine ANGPTL8 induces CCL5 secretion. Macrophages are the predominant source of CCL5, which is secreted in response to various proinflammatory stimuli (14). We demonstrated that rA8 increased the CCL5 production of macrophages. CCL5 contributes to the infiltration of circulating monocytes into the peripheral tissue that plays a pivotal role in the development and maintenance of obesity-associated chronic low-grade inflammation. Our study revealed that the ANGPTL8-induced postprandial increase of monocytes was mediated by CCL5-CCR5 axis. Blockage of CCR5 significantly reduced the metabolic inflammation and insulin resistance in Angptl8-overexpressing mice. Therefore, we identified that ANGPTL8 regulates food-driven monocyte rhythms by promoting CCL5 production.

Our research elucidates a mechanism by which ANGPTL8 promotes metabolic inflammation through a CCL5-CCR5–dependent pathway. It is important to distinguish this pathway from the previously reported roles of ANGPTL8 in liver-specific (8) and neuro-specific inflammation (9). The pleiotropic effects of ANGPTL8 appear to be context dependent, engaging distinct pathways across various tissues to mediate unique pathological processes. Our identification of ANGPTL8’s role in directly regulating a classic chemokine axis underscores its importance as a central node linking metabolic stress to innate immune activation and broadens the potential therapeutic strategies for targeting ANGPTL8-related pathologies. Moreover, we demonstrate that this pathogenic mechanism is exclusive to ANGPTL8, emphasizing its nonredundant and specific role in metabolic inflammation, and reinforcing its status as a unique therapeutic target.

The proinflammatory capacity of ANGPTL8 appears to function through mechanisms that are largely independent of its hyperlipidemic effects. This is most clearly demonstrated by our experiment involving fenofibrate, where pharmacological normalization of triglyceride levels did not reduce ANGPTL8-induced monocytosis. Although triglyceride levels were not assessed following ANGPTL8 administration in the ANGPTL3-knockdown model, the observation that ANGPTL8 significantly increased monocyte levels in the context of markedly low baseline triglycerides provides additional genetic evidence supporting this model of pathway dissociation. Collectively, these distinct pharmacologic and genetic approaches reinforce the conclusion that ANGPTL8 can promote inflammation through pathways not strictly reliant on concurrent triglyceride elevation.

Furthermore, the essential role of this specific pathway is underscored by our intervention studies. The blunted inflammatory response to refeeding in *Angptl8^HepOE^* mice on HFD likely reflects a ceiling effect, wherein fasting inflammation is already maximally elevated. The consistent upregulation of key proinflammatory cytokines (IL1B, IL6, TNFA) across our in vivo models indicates these factors constitute an essential inflammatory module downstream of the primary CCL5-CCR5 axis. We propose that these cytokines are amplified as part of the inflammatory cascade initiated by the primary CCL5-CCR5 axis, thereby contributing to the systemic amplification of the immune response. Crucially, the subsequent amelioration of this phenotype and the metabolic deficits through CCR5 inhibition demonstrate that the pathogenic effects of ANGPTL8 are mechanistically dependent on CCR5 signaling. This genetic and pharmacologic evidence solidifies a linear pathway where nutritional stress-induced ANGPTL8 activates CCR5 to drive systemic inflammation and insulin resistance.

It has been speculated for a long time that hepatocytes might modulate monocyte hematopoiesis and egress (28, 29). As the largest metabolic organ, the liver sensitively responds to the food signals and secretes hepatokines, leading to the robust regulation of metabolic and immune processes. Here, we give evidence that a hepatokine named ANGPTL8 controls monocyte egress to the blood circulation, that ANGPTL8 is involved in the crosstalk between liver and immune cells in circulation, and that it mediates food-driven monocyte dynamics. Although many chemokines can regulate the recruitment of monocytes, few of them induce food-driven rhythms of changes in serum levels. ANGPTL8 was identified as a unique chemokine with food-entrained rhythms of expression, which we believe points to its important role in food-driven monocyte dynamics.

Our study establishes ANGPTL8 as a key nutritional checkpoint that promotes monocyte expansion during refeeding through a direct signaling mechanism, as evidenced by the ability of recombinant ANGPTL8 to directly stimulate macrophage CCL5 production and monocyte migration in vitro, an effect that was abolished by CCR5 inhibition and occurred independently of systemic metabolic alterations, endotoxemia, or changes in nutrient utilization. This ANGPTL8-CCL5-CCR5 pathway operates independently of both the canonical ANGPTL3/8 lipid metabolic complex and the fasting-induced AMPK/PPARA-CCL2 axis described by Jordan et al. (4), revealing complementary hepatic pathways that oppositely regulate immune responses according to nutritional state. While our study emphasizes a liver-derived endocrine signal and Jordan et al. focus on bone marrow precursor energetics, these models collectively highlight the complexity of nutritional immunomodulation. Importantly, we demonstrate that this proinflammatory pathway exacerbates obesity-related metabolic dysfunction, identifying the ANGPTL8-CCL5-CCR5 axis as a promising therapeutic target for combating metabolic inflammation and its associated complications.

Overall, our study enhances our understanding of ANGPTL8, identifying roles for it in metabolic inflammation and the regulation of monocyte dynamics. We have thus identified ANGPTL8 as a key regulator of the circulating monocytes in response to food.

## Methods

### Sex as a biological variable.

All experiments in this study were performed using age-matched adult male mice. The use of male mice was to eliminate potential confounding effects of the estrous cycle and associated hormonal fluctuations on metabolic and inflammatory parameters.

### Mouse strains used in the study.

The mice were given free access to food and water and housed in a temperature-controlled environment (23°C ± 2°C) with a 12-hour light/dark cycle. Wild-type C57BL/6J mice were from Beijing Huafukang Bioscience Co. Ltd. Hepatocyte-specific *Angptl8*-overexpression (*Angptl8^HepOE^*) mice were generated by Cyagen Biosciences Inc. Hepatocyte-specific *Angptl8*-knockout (*Angptl8^HepKO^*) mice on a C57BL/6J genetic background were generated by Model Animal Research Center of Nanjing University (Nanjing, China) using CRISPR/Cas9-mediated genome engineering. For in vivo knockdown of ANGPTL3, 8-week-old C57BL/6J mice were intravenously injected with AAV8 vectors expressing short hairpin RNA targeting *Angptl3* (AAV8-*shAngptl3*, 5′-GCAGCTAACCAACTTAATTC-3′) or control scrambled shRNA (AAV8-*shScramble*) obtained from Gene Pharma.

Metabolic inflammation was induced by feeding 6-week-old male C57BL/6J mice with a HFD (60 kcal% fat, 20 kcal% carbohydrate, and 20 kcal% protein, Medicience Ltd., catalog MD12033) for 3 months. Control mice were maintained with a normal diet (10 kcal% fat, 70 kcal% carbohydrate, and 20 kcal% protein, Jiangsu Xietong, Inc., catalog SWS9102). For pharmacological normalization of triglycerides, *Angptl8^HepOE^* mice and their *Loxp* littermates were fed a chow diet supplemented with 200 mg/kg fenofibrate for 2 weeks, following an established protocol (30).

### Experimental animal fasting and refeeding.

For overnight fasting, food was removed at 9 pm (ZT2), and the cage bedding and nesting material were changed to prevent coprophagy. For refeeding, mice were given food at 9 am (ZT14) and refeeding lasted for 2 hours; meanwhile, fasting mice were still under food withdraw. Fasting and refeeding mice had access to water ad libitum. All fasting/refeeding experiments were performed using this protocol.

### Cell lines.

The human monocytic cell lines U937 and THP-1 were obtained from the ATCC. Cell line identities were authenticated by short tandem repeat profiling. Both cell lines were routinely tested for mycoplasma contamination using the MycoAlert Mycoplasma Detection Kit (Lonza Bioscience, catalog LT07-318), and all experiments were conducted with mycoplasma-free cultures.

### Human MDMs.

Human PBMCs were isolated from whole blood of healthy donors by standard Ficoll density gradient centrifugation. Monocytes were subsequently purified from PBMCs using a human monocyte isolation kit (Solarbio, catalog P5290) following the manufacturer’s instructions. To generate MDMs, the purified monocytes were cultured in RPMI-1640 medium supplemented with 10% FBS, 1% penicillin-streptomycin, and 50 ng/mL recombinant human M-CSF (Abcam, catalog ab62015) for 6 days, with fresh medium containing M-CSF replaced on day 3.

### Flow cytometry.

Cell suspensions were stained with appropriate antibodies for 30 minutes on ice. Commercial antibodies used in this study included anti-mouse FITC-CD45 (BioLegend, catalog 103108), Percp/Cy5.5-CD11b (BioLegend, #101228), and APC-Ly6C (BioLegend, catalog 128016). All antibodies were diluted according to the manual from the manufacturer’s website. Dead cells and doublets were removed by dead-cell dye staining (Zombie Aqua Fixable Viability Kit, BioLegend, catalog 423102).

### Liver immunohistochemistry.

The paraffin-embedded sections were deparaffinized and rehydrated. The sections were incubated with primary antibodies against CD68 (Servicebio, catalog GB113109) for 2 hours at room temperature and washed 3 times with PBS. Then, the sections were incubated with horseradish peroxidase–conjugated secondary antibodies (Servicebio, catalog GB23303) for 1 hour at room temperature, followed by 3,3′-diaminobenzidine development, hematoxylin staining, dehydration, and mounting. The stained sections were observed using light microscopy. Staining intensity per HMF in 8 fields per section was determined by ImageJ (NIH).

### Isolation and differentiation of BMDMs.

Bone marrow cells were obtained by flushing the femurs and tibias from 6- to 8-week-old male mice using a 1 mL syringe, passed through a 100 μm cell strainer, and centrifuged at 500*g* for 5 minutes at 4°C. The pellet was suspended in 500 μL of red blood cell lysis buffer (Servicebio, catalog G2015-500 ML), incubated for 15 seconds to remove red blood cells, and centrifuged at 500*g* for 5 minutes at 4°C. The pellet was suspended and cultured in DMEM (HyClone, catalog SH30021.01) supplemented with 10 ng/mL macrophage colony-stimulating factor (M-CSF, PeproTech, catalog 315-02), 10% FBS and 1% penicillin-streptomycin. After 3 days of differentiation, the culture medium was refreshed, and BMDMs were continuously cultured with DMEM containing 10 ng/mL M-CSF for another 2 days before experimentation.

### rA8 treatment.

Before rA8 treatment, cells were serum starved for 12 hours. Then, the BMDMs were stimulated with 40 nM rA8 protein (Cusabio, catalog CSB-MP844436MO) or vehicle (PBS) for an additional 24-hour period. All the recombinant proteins were tested by SDS-PAGE quantitative densitometry by Coomassie blue staining, and the purities were 95%–98%. No aggregated or sticky protein was found. Proteins were reconstituted in deionized sterile water to a concentration of 0.1–1.0 mg/mL, 5% glycerol (final concentration) was added, and proteins were aliquoted for long-term storage at –20°C/–80°C.

### Macrophage polarization and treatment.

BMDMs were first polarized by treating them for 24 hours with LPS (100 ng/mL) plus murine IFNG (20 ng/mL) to generate M1 macrophages or with murine IL4 (20 ng/mL) to generate M2 macrophages. Cells treated with medium alone were defined as M0 macrophages. After the polarization period, cells were washed twice with PBS to remove polarizing cytokines and then incubated in fresh medium containing recombinant ANGPTL8 (rA8) at the indicated concentrations for an additional 24 hours.

### Chemokine receptor antagonists.

The CCR5 antagonist TAK-220 (MedChem Express, catalog HY-19974) was dissolved in DMSO and diluted in saline to achieve a maximal DMSO concentration of 1% and was applied daily by oral gavage. For rA8 injection, 4 mg/kg TAK-220 was administered by oral gavage 3 hours before rA8 injection. For HFD feeding, 4 mg/kg/d TAK-220 was administered by oral gavage for 4 weeks after 12-week-HFD feeding. In vitro, 10 nM TAK-220 was added to medium to inhibit migration. The CCR7 antagonist SLW131 (10 nM, MedChem Express, catalog HY-173052), CCR8 antagonist R243 (10 nM, MedChem Express, catalog HY-122219), CCL12 inhibitor AMR69 (10 nM, MedChem Express, catalog HY-B0673), CXCR2 antagonist SB225002 (10 nM, MedChem Express, catalog HY-16711), CXCR3 antagonist AMG487 (10 nM, MedChem Express, catalog HY-15319), and anti-CXCL9 antibody MIG-2F5.5 (10 nM, MedChem Express, catalog HY-P990255) were dissolved and diluted according to the manufacturer’s instructions and were added to medium to inhibit migration.

### Signaling pathway inhibitors.

In vitro, cells were pretreated with inhibitors of ERK (U0126; 10 nM, MedChem Express, catalog HY-12031) and P38 (SB203580; 10 nM, MedChem Express, catalog HY-10256) for 30 minutes before incubation with rA8 (40 nM) for 24 hours. In vivo, the P38 inhibitor SB203580 and ERK inhibitor U0126 were dissolved in DMSO and diluted in saline to achieve a maximal DMSO concentration of 1% and were injected (5 mg/kg for SB203580; 10 mg/kg for U0126, i.p.) 3 hours before rA8 injection.

### Transwell migration assay.

Primary mouse monocytes (5 × 10^5^) were seeded with 100 μL serum-free DMEM atop 8 μm (5 μm for U937 and THP-1 cell) polycarbonate filter inserts in Transwell chamber plates (Corning, catalog 3422), and 500 μL serum-free DMEM was added to the lower chamber. PBS or 10 nM TAK-220 was added to the upper chamber. After incubation for 30 minutes, conditional media collected from scramble- or rA8-treated BMDMs was replaced to the lower chamber. After incubation for 24 hours, cells that had migrated to the lower chamber were counted by flow cytometry. For representative imaging, parallel experiments were performed. In these cases, the Transwell inserts were processed after incubation: the nonmigrated cells on the upper surface were removed by swabbing, and the migrated cells on the lower surface were fixed with 4% PFA for 20 minutes and stained with 0.1% crystal violet for 15 minutes.

### ELISA.

Commercially available mouse ELISA kits were used to measure ANGPTL8 (EIAab Science, catalog E11644m for mouse; EIAab Science; catalog E11644h for human), mouse interleukin (IL6) (Elabscience Biotechnology Co. Ltd., catalog E-EL-M0044c), mouse tumor necrosis factor (TNFA) (Elabscience Biotechnology Co. Ltd., catalog E-EL-M0049c), mouse IL1B (Elabscience Biotechnology Co. Ltd., catalog E-EL-M0037c), and mouse CCL5 (Elabscience Biotechnology Co. Ltd., catalog E-EL-M0009) levels in the serum or in the cell supernatants according to the manufacturer’s instructions.

### RNA isolation and real-time quantitative PCR.

Total RNA was extracted using TRIzol (Thermo Fisher Scientific, catalog 15596018) following the manufacturer’s instructions and quantified through use of a Nanodrop. To prepare RNA for PCR analysis, 1 μg total RNA was converted to cDNA using Hifair II 1st Strand cDNA Synthesis SuperMix (Yeasen, catalog 11120ES60). Real-time quantitative PCR was performed using the qPCR Hieff UNICON qPCR SYBR Green Master Mix (Low Rox) (Yeasen, catalog 11199ES08) and primers for mouse *Angptl8* (5′-CTCAATGGCGTGTACAGAGC, 3′-TCGAAGGTGTAAAGCGTCCT), *Ccl5* (5′-GTGCCCACGTCAAGGAGTAT, 3′-CTCTGGGTTGGCACACACTT), and *Gapdh* (5′-AGGTCGGTGTGAACGGATTTG, 3′-TGTAGACCATGTAGTTGAGGTCA) under the following conditions: initial denaturation at 95°C for 30 seconds, 40 cycles at 95°C for 10 seconds, 60°C for 30 seconds. Each sample was analyzed in triplicate. Relative mRNA levels were calculated using the 2^–ΔΔCt^ method and normalized to *Gapdh* mRNA levels.

### Western blotting.

Cells were washed with PBS 3 times and collected with RIPA lysis buffer (Beyotime, catalog P0013B) according to the manufacturer’s protocol. Equal amounts of protein (20 μg) were loaded into each lane, separated through 10% SDS polyacrylamide gel electrophoresis, and electrotransferred to PVDF membranes. The PVDF membranes were blocked in blocking buffer for 2 hours at room temperature, followed by incubation with the following antibodies overnight at 4°C: mouse anti-ACTB (Proteintech, catalog 66009-1-Ig); rabbit anti-ERK (Cell Signaling Technology, catalog 4695T), anti-phospho-ERK (Thr202/Tyr204; Cell Signaling Technology, catalog 4370T), anti-P38-MAPK (Cell Signaling Technology, catalog 8690T), anti-phospho-P38-MAPK (Thr180/Tyr182; Cell Signaling Technology, catalog 4511T), anti-JAK1 (Cell Signaling Technology, catalog 3344T), anti-phospho-JAK1 (Cell Signaling Technology, catalog 3331S), anti-STAT1 (Cell Signaling Technology, catalog 14994T), and anti-phospho-STAT1(Cell Signaling Technology, catalog 9167S). The blots were subsequently rinsed with TBST 3 times and incubated with anti-mouse, anti-rabbit, or anti-rat peroxide-conjugated secondary antibodies for 90 minutes. The protein bands were visualized and detected using an enhanced chemiluminescence system.

### RNA sequencing and bioinformatic analysis.

Total RNA was isolated from samples using TRIzol reagent (Thermo Fisher Scientific, catalog 15596018) following the manufacturer’s instructions. RNA integrity and concentration were verified prior to library construction. Sequencing libraries were prepared and sequenced on an Illumina platform by Shanghai Genminix Informatics Co. Ltd. Raw sequencing reads underwent quality control assessment (e.g., using FastQC) and adapter trimming. Differential gene expression analysis was performed using the limma R package (v3.44.3). Genes with an adjusted *P* value < 0.05 and absolute log_2_ fold change > 1 were considered significantly differentially expressed. All statistical analyses and visualizations were conducted using R Studio (version 1.1.442).

### Statistics.

Data are presented as mean ± SEM. Comparisons between 2 groups were analyzed using a 2-tailed Student’s *t* test. For comparisons among more than 2 groups, 1-way or 2-way ANOVA was applied followed by Tukey’s test. A *P* value of less than 0.05 was considered statistically significant.

### Study approval.

All animal experiments were approved by the Institutional Animal Care and Use Committee of the Institute of Model Animal of Tongji Hospital, Huazhong University of Science and Technology (approval protocol no. TJH-202206002), and were conducted in accordance with the relevant ethical guidelines. The animals received humane care according to the criteria outlined in the *Guide for the Care and Use of Laboratory Animals* (National Academies Press, 2011) prepared by the National Academy of Sciences and published by the NIH.

All procedures involving humans were approved by the Ethical Committee of Tongji Hospital, Tongji Medical College, Huazhong University of Science and Technology (IRB ID TJ-C20220801) and conducted in accordance with the principles of the Declaration of Helsinki. Written informed consent was obtained from all participants.

### Data availability.

The raw RNA-seq data generated in this study have been deposited in the NCBI Sequence Read Archive (SRA) database under the Bioproject accession number (PRJNA992231). All supporting data values are provided in the Supporting Data Values file. All other data supporting the findings of this study are available within the article and its supplemental information files.

## Author contributions

Conceptualization: RRK, SYW, DPL, and XFY. Methodology: RRK and LH. Investigation: SYW and XYM. Data curation: XYM, LH, YXX, BBM, HJZ, YMG, LMP, and PQL. Supervision: DPL and XFY. Writing of the original draft: RRK and SYW. Review and editing of the manuscript: YXX, BBM, HJZ, YMG, LMP, PQL, YY, ZLL, DLM, WJL, YC, DPL, and XFY.

## Funding support

- National Natural Science Foundation of China grant 82270880, 82470907, 81974109, and 81570740.
- Young Scientists Fund of the National Natural Science Foundation of China grant 82400944.

## Supplementary Material

Supplemental data

Unedited blot and gel images

Supporting data values

## Version 1 11/25/2025

In-Press Preview

## Version 2 01/09/2026

Electronic publication
