# Supplementary material for: ANGPTL8 links refeeding to monocyte dynamics and metabolic inflammation via the CCL5-CCR5 axis
Source: JCI Insight. 2025 Nov 25;11(1):e196605. doi: 10.1172/jci.insight.196605 (PMC12890512; doi:10.1172/jci.insight.196605)
Supplement: Supplemental data [file jciinsight-11-196605-s049.pdf]

Fig. S1

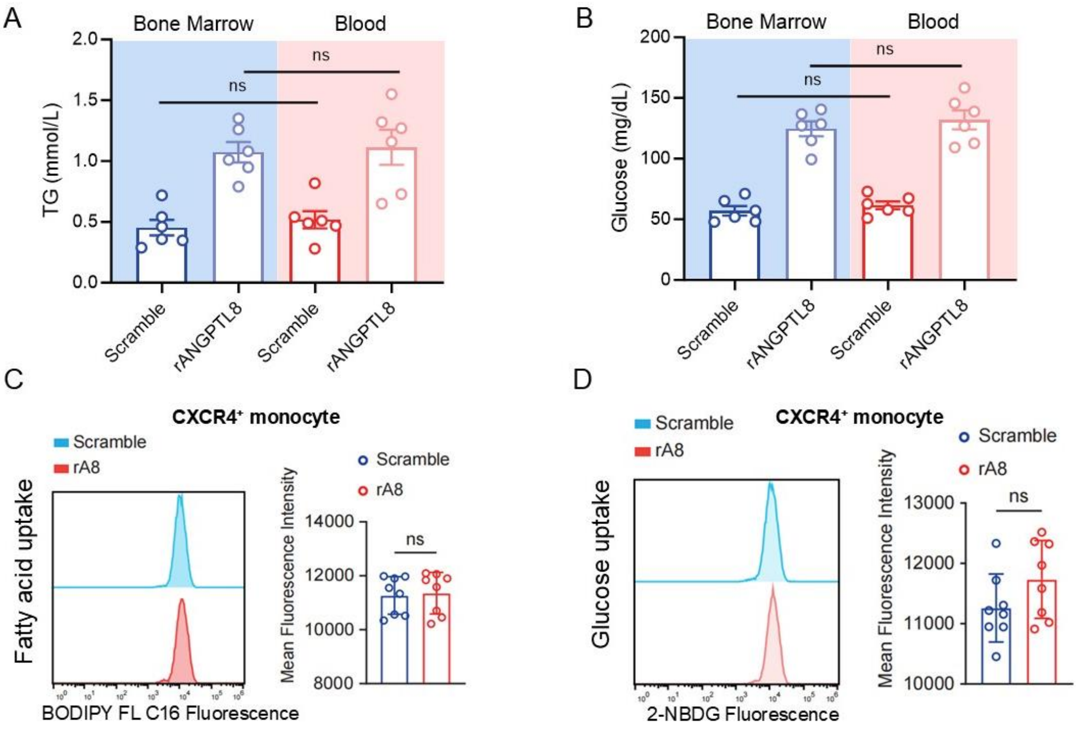

Figure S1. ANGPTL8 does not alter nutrient availability in the bone marrow microenvironment or nutrient uptake in monocyte precursors.

(**A**) Triglyceride and (**B**) glucose concentrations were measured in paired samples of plasma and bone marrow flush fluid (n = 6 mice per group). (**C**) Representative flow cytometry histograms and quantitation of MFI of BODIPY FL C16 in CXCR4$^+$ monocytes (n = 8 mice per group). (**D**) Representative flow cytometry histograms and quantitation of MFI of 2-NBDG in CXCR4$^+$ monocytes. All samples are biologically independent replicates and n indicates the number of biologically independent samples examined. Data are mean ± SEM (n = 8 mice per group). Statistical significance was determined by paired two-tailed t-test (**A, B**) or unpaired two-tailed t-test (**C, D**). ns, not significant. rA8, recombinant ANGPTL8; MFI, mean fluorescence intensity.

Fig. S2

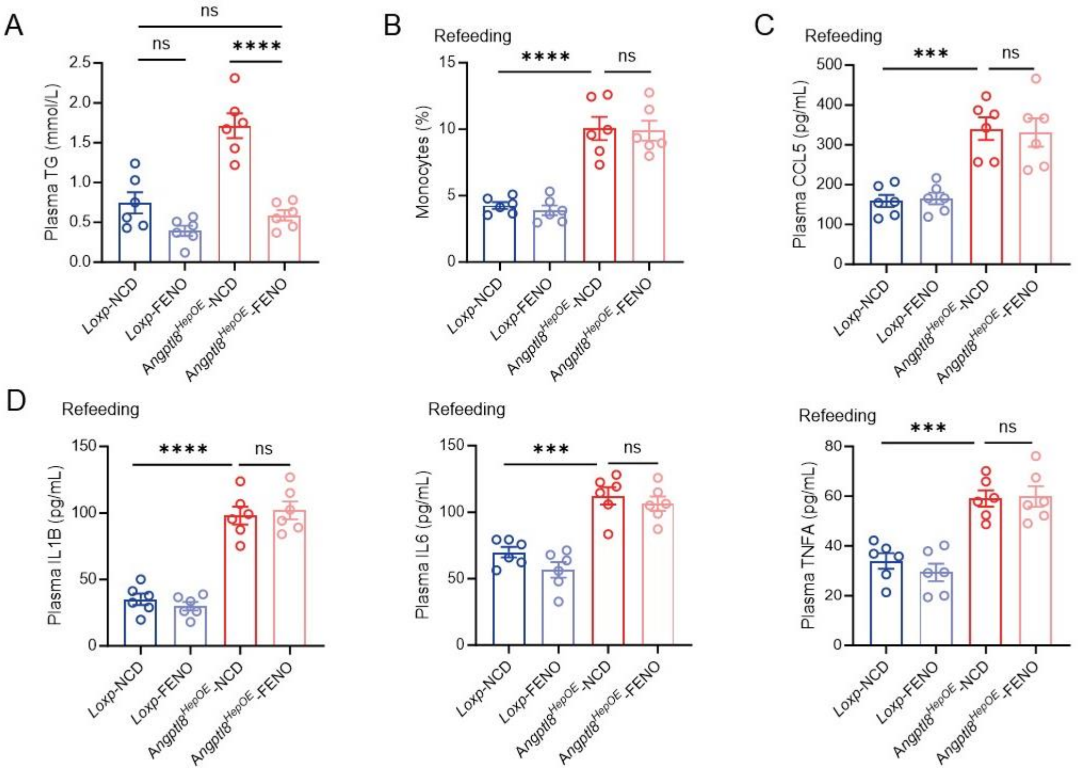

Figure S2. ANGPTL8-driven monocytosis and inflammation are independent of its hyperlipidemic effect.

(**A**) Plasma TG levels in *Angptl8*^{HepOE} mice and *LoxP* littermate controls after two weeks of treatment with fenofibrate (200 mg/kg in chow) or control chow (n = 6 mice per group). (**B**) Circulating monocyte populations and (**C**) Plasma CCL5 levels in the same groups (n = 6 mice per group). (**D**) Plasma pro-inflammatory cytokines in the indicated groups (n = 6 mice per group). All samples are biologically independent replicates and n indicates the number of biologically independent samples examined. Data are mean ± SEM. Statistical significance was determined by one-way ANOVA with Tukey's test. ns, not significant; * $p < 0.05$, ** $p < 0.01$, *** $p < 0.001$, **** $p < 0.0001$. TG, triglyceride; NCD, normal chow diet; FENO, fenofibrate.

Fig. S3

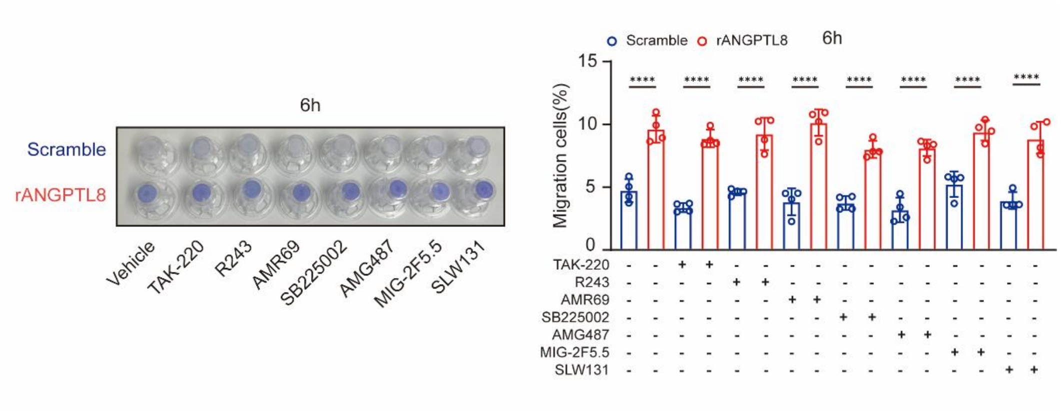

Figure S3. Effects of chemokine inhibitors on the 6-hour ANGPTL8-induced migration of BMDMs.

Representative image and quantification of BMDMs migration with rA8 over time (n = 3 independent experiments). TAK-220 CCR5 antagonist, R243 CCR8 antagonist, AMR69 CCL12 inhibitor, SB225002 CXCR2 antagonist, AMG487 CXCR3 antagonist, MIG-2F5.5 anti-CXCL9 antibody, SLW131 CCR7 antagonist. Migrated cells were fixed and stained with 0.1% crystal violet**.** The data are shown as the mean ± SEM. and were statistically analyzed by one-way ANOVA. All samples are biologically independent replicates and n indicates the number of biologically independent samples examined. All the p values were two-sided and adjustments were made for multiple comparisons. * $p < 0.05$, ** $p < 0.01$, *** $p < 0.001$, **** $p < 0.0001$. BMDMs bone marrow-derived macrophages.

Fig. S4

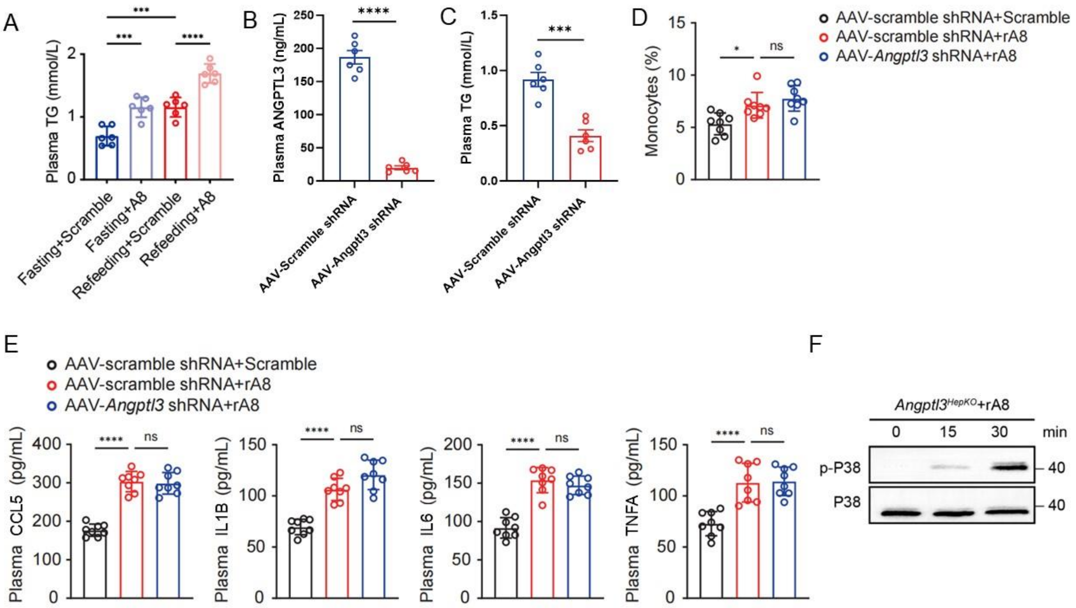

Figure S4. ANGPTL8 induces inflammation independently of ANGPTL3.

(**A**) Plasma triglyceride levels in mice under fasting or refed conditions after injection with scramble or recombinant ANGPTL8 (n = 6 mice per group). (**B**) Plasma ANGPTL3 levels and (**C**) plasma triglyceride levels in *Angptl3* knockdown mice (n = 6 mice per group). (**D**) Circulating monocyte populations in mice injected with AAV8-*shAngptl3* and recombinant ANGPTL8 compared to the scramble control group (n = 8 mice per group). (**E**) Plasma levels of CCL5 and pro-inflammatory cytokines of indicated groups. (**F**) Phospho-P38 and total P38 protein levels in BMDMs. All samples are biologically independent replicates and n indicates the number of biologically independent samples examined. Data are mean ± SEM. Statistical significance was determined by (**A**) two-way ANOVA with Tukey's test, (**B, C**) unpaired two-tailed t-test, and (**D, E**) one-way ANOVA with Tukey's multiple-comparison test. ns, not significant; * $p < 0.05$, ** $p < 0.01$, *** $p < 0.001$, **** $p < 0.0001$. TG, triglyceride; rA8, recombinant ANGPTL8.

Fig. S5

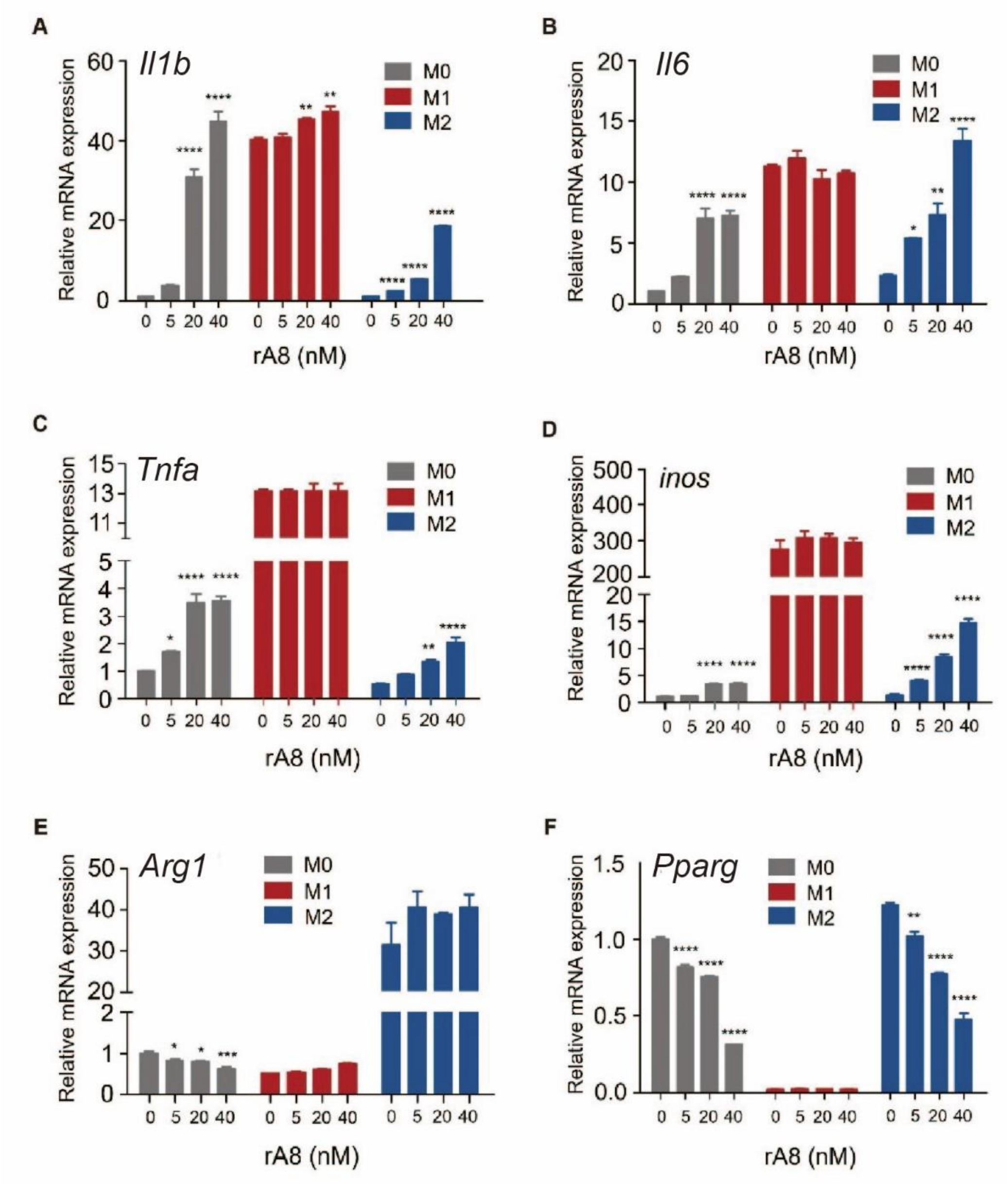

Figure S5. ANGPTL8 differentially regulates inflammatory gene expression in pre-polarized macrophage subsets.

(**A-D**) mRNA expressions of M1 marker genes (including *Il1b*, *Il6*, *Tnfa*, and *inos*) in pre-polarized BMDMs with rA8 at different doses (n = 6 cells examined over three independent experiments). (**E, F**) mRNA expressions of M2 marker genes (including *Arg1* and *Pparg*) in pre-polarized BMDMs with rA8 at different doses (n = 6 cells examined over three independent experiments). The data are shown as the mean ± SEM. and were statistically analyzed by two-way ANOVA. All samples are biologically independent replicates and n indicates the number of biologically independent samples examined. All the p values were two-sided and adjustments were made for multiple comparisons. * $p < 0.05$, ** $p < 0.01$, *** $p < 0.001$, **** $p < 0.0001$. BMDMs bone marrow-derived macrophages, M0 initial macrophages, M1 proinflammation macrophages, M2 anti-inflammation macrophages.

Fig. S6

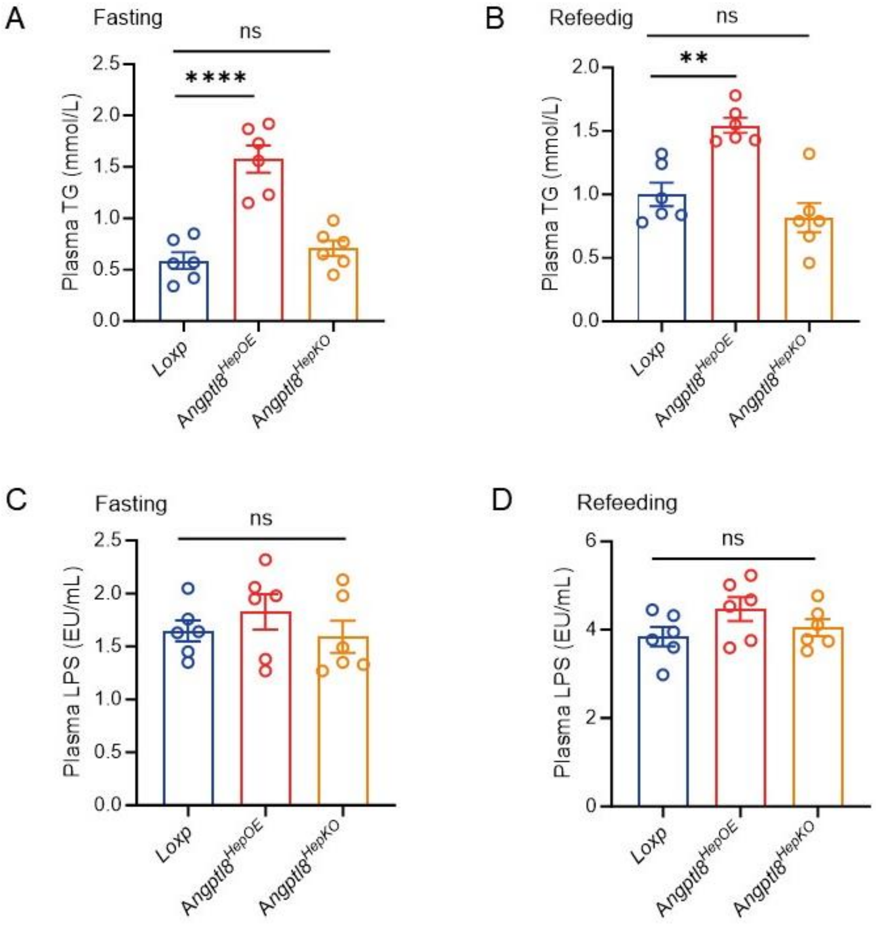

Figure S6. Hepatocyte-specific manipulation of *Angptl8* alters postprandial triglyceridemia without affecting plasma endotoxin levels.

(**A, B**) Plasma triglyceride (TG) levels in *Angptl8^{HepOE}* mice, *Angptl8^{HepKO}* mice and *LoxP* controls under (**A**) fasting and (**B**) refeeding conditions (n = 6 mice per group). (C, D) Plasma LPS levels in the indicated groups under (**C**) fasting and (**D**) refeeding conditions (n = 6 mice per group). All samples are biologically independent replicates and n indicates the number of biologically independent samples examined. Data are presented as mean ± SEM. Statistical significance was determined by one-way ANOVA with Tukey's multiple-comparison test. ns, not significant; * $p < 0.05$, ** $p < 0.01$, *** $p < 0.001$, **** $p < 0.0001$. TG, triglyceride; LPS, lipopolysaccharides.

Fig. S7

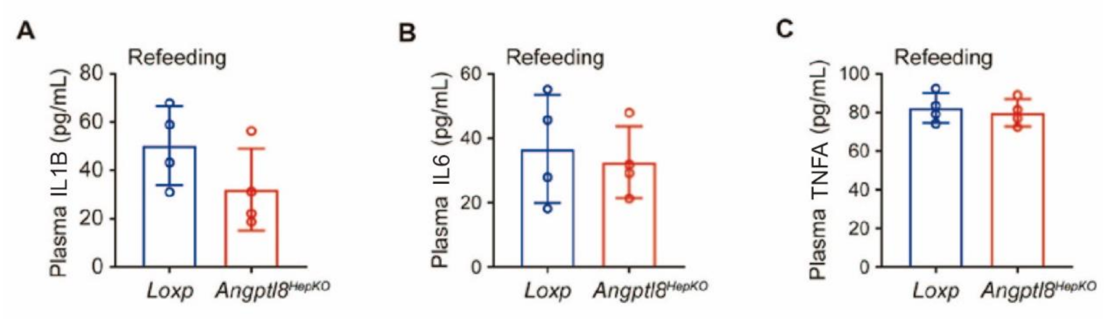

Fig. S7. ANGPTL8 did not impact proinflammation chemokines in refeeding mice with normal diet.

(**A-C**) Plasma proinflammation cytokines levels of refeeding mice with normal diet (n = 4 mice per group). All samples are biologically independent replicates and n indicates the number of biologically independent samples examined. The data are shown as the mean ± SEM and were statistically analyzed by two-tailed Student's test. All samples are biologically independent replicates and n indicates the number of biologically independent samples examined. All the p values were two-sided. * $p < 0.05$, ** $p < 0.01$, *** $p < 0.001$, **** $p < 0.0001$.

Fig. S8

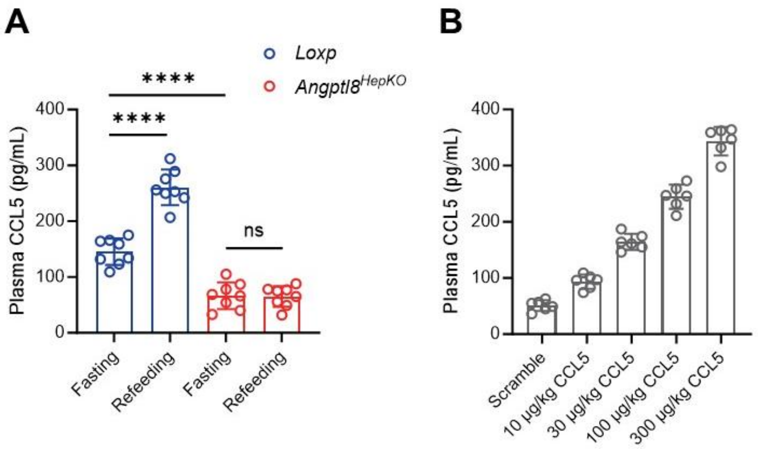

Figure S8. ANGPTL8 deficiency abolishes refeeding-induced CCL5 upregulation and determination of recombinant CCL5 dose.

(**A**) Plasma CCL5 protein levels in *Loxp* and *Angptl8*^{HepKO} mice under fasted and refed conditions (n = 8 mice per group). (**B**) Plasma CCL5 levels following intraperitoneally injection of increasing doses of CCL5 protein (n = 6 mice per group). Data are presented as mean ± SEM and were statistically analyzed by one-way ANOVA with Tukey's multiple-comparison test (**A**). All samples are biologically independent replicates and n indicates the number of biologically independent samples examined. All the p values were two-sided and adjustments were made for multiple comparisons. * $p < 0.05$, ** $p < 0.01$, *** $p < 0.001$, **** $p < 0.0001$.

Fig. S9

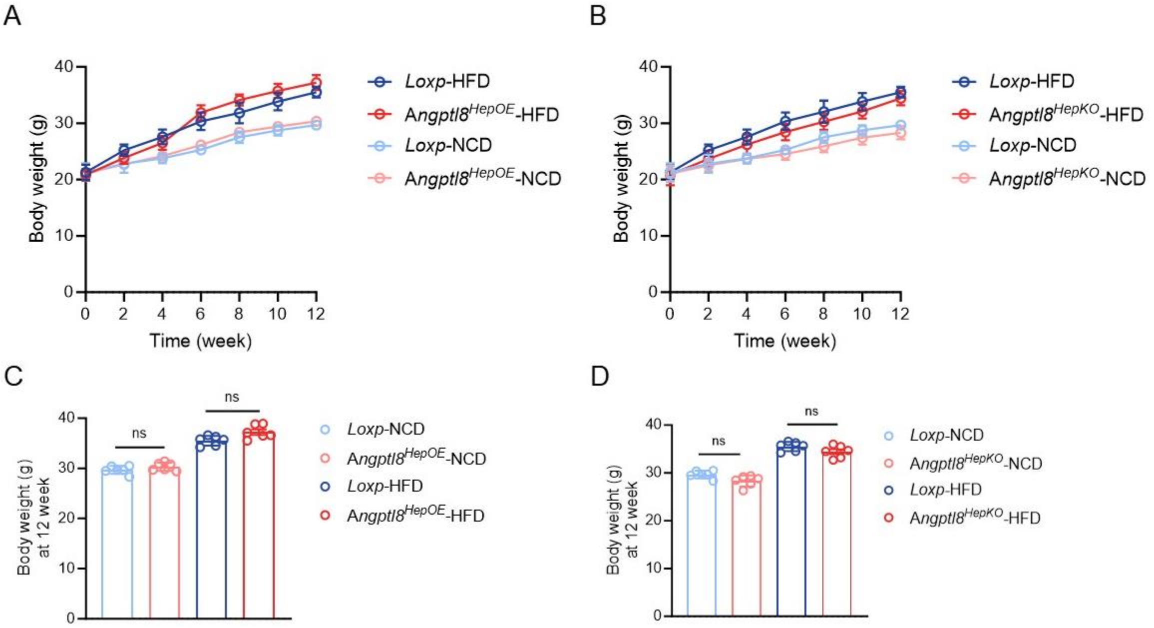

Figure S9. Body weight gain in *Angptl8*^{HepOE} and *Angptl8*^{HepKO} mice under normal and high-fat feeding.

(**A**) Body weight growth curves of *Angptl8*^{HepOE} mice (n = 6 mice per group). (**B**) Body weight growth curves of *Angptl8*^{HepKO} mice (n = 6 mice per group). (**C**) Final body weight of *Angptl8*^{HepOE} mice and *Loxp* controls measured at the endpoint of the 12-week feeding period. (**D**) Final body weight of *Angptl8*^{HepKO} mice and *Loxp* controls measured at the endpoint of the 12-week feeding period. All samples are biologically independent replicates and n indicates the number of biologically independent samples examined. Data are presented as mean ± SEM. Statistical analysis was performed by two-way ANOVA with Tukey's multiple-comparison test (**C, D**). HFD, high-fat diet; NCD, normal chow diet.
