# Supplementary material for: ANGPTL8 links refeeding to monocyte dynamics and metabolic inflammation via the CCL5-CCR5 axis
Source: JCI Insight. 2025 Nov 25;11(1):e196605. doi: 10.1172/jci.insight.196605 (PMC12890512; doi:10.1172/jci.insight.196605)
Supplement: Unedited blot and gel images [file jciinsight-11-196605-s050.pdf]

Fig. 4B P38 full blot

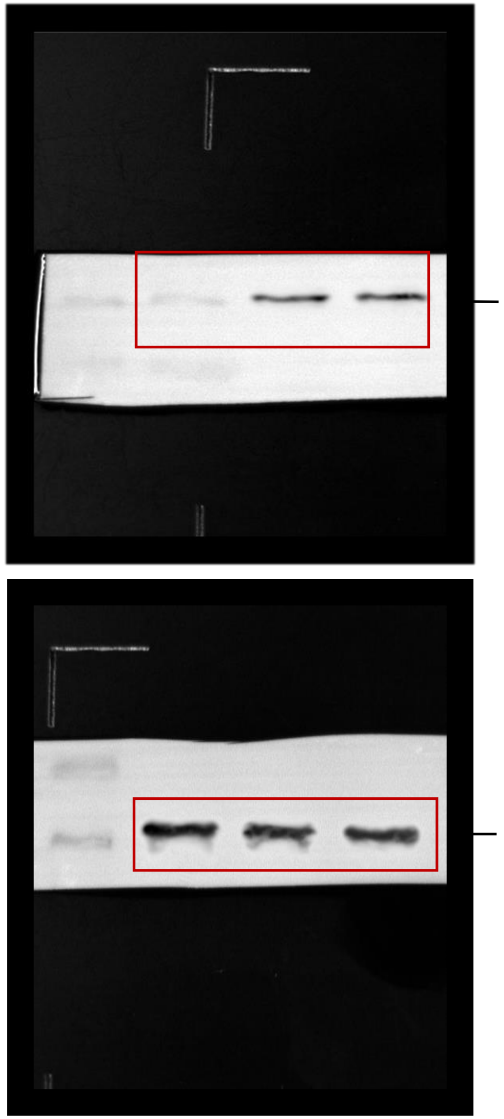

The red box indicates the region used in the final figure. Membranes were probed sequentially for target proteins and loading controls as indicated. Molecular weight markers are shown for reference.

Fig. 4B ERK full blot

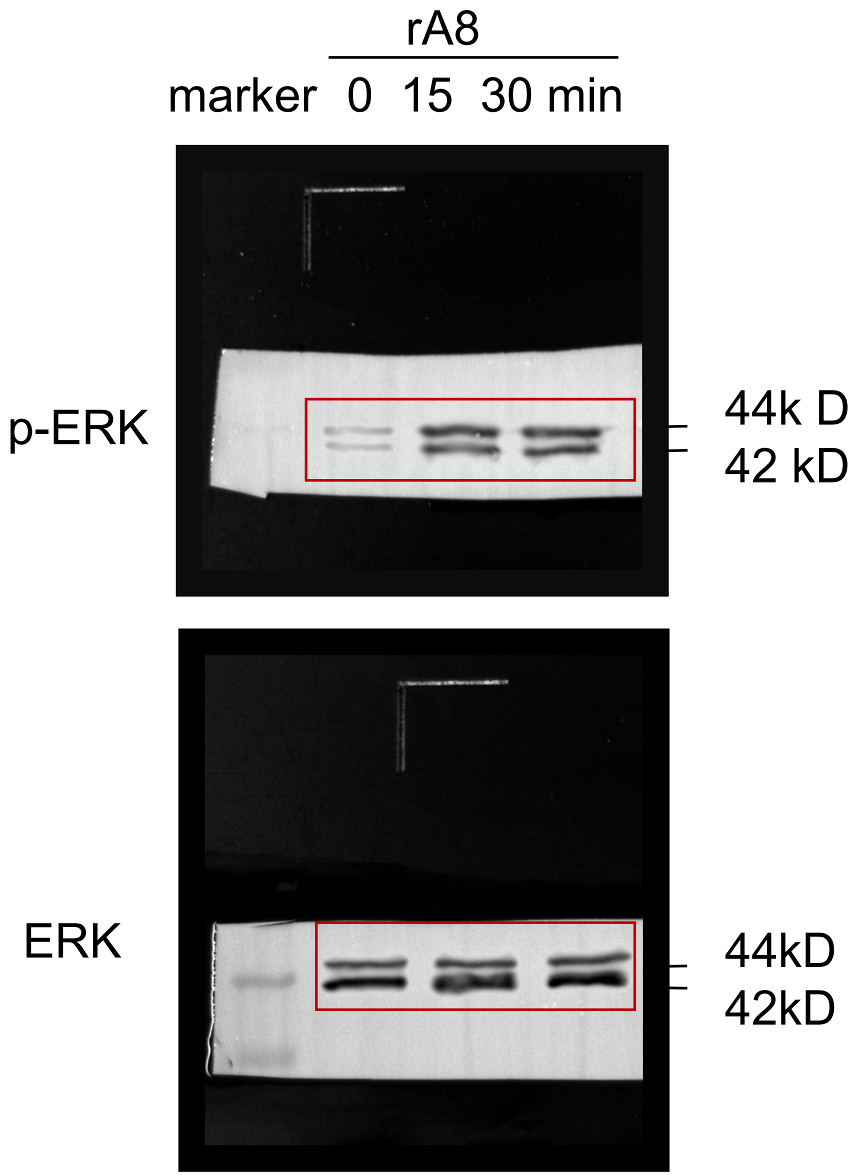

The red box indicates the region used in the final figure. Membranes were probed sequentially for target proteins and loading controls as indicated. Molecular weight markers are shown for reference.

Fig. 4B JAK1 full blot

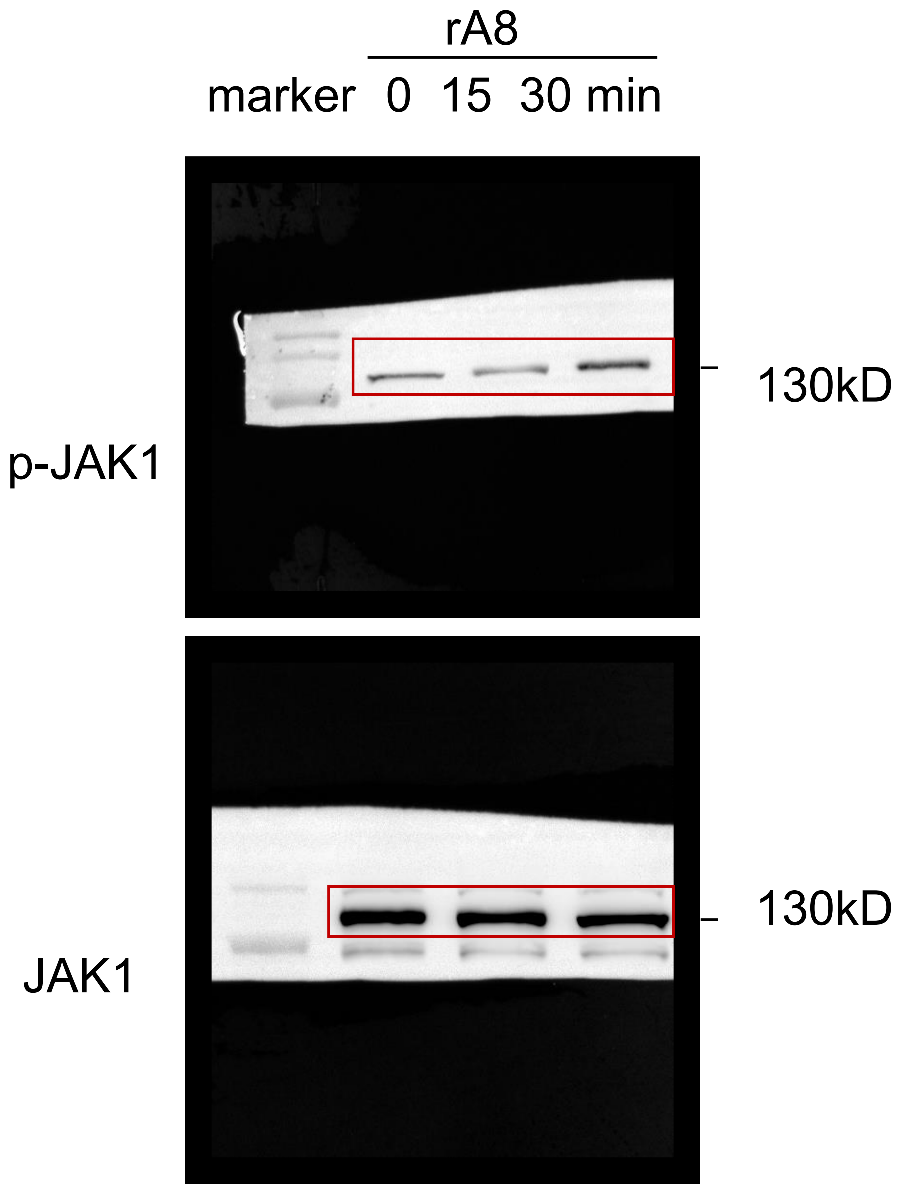

The red box indicates the region used in the final figure. Membranes were probed sequentially for target proteins and loading controls as indicated. Molecular weight markers are shown for reference.

Fig. 4B STAT1 full blot

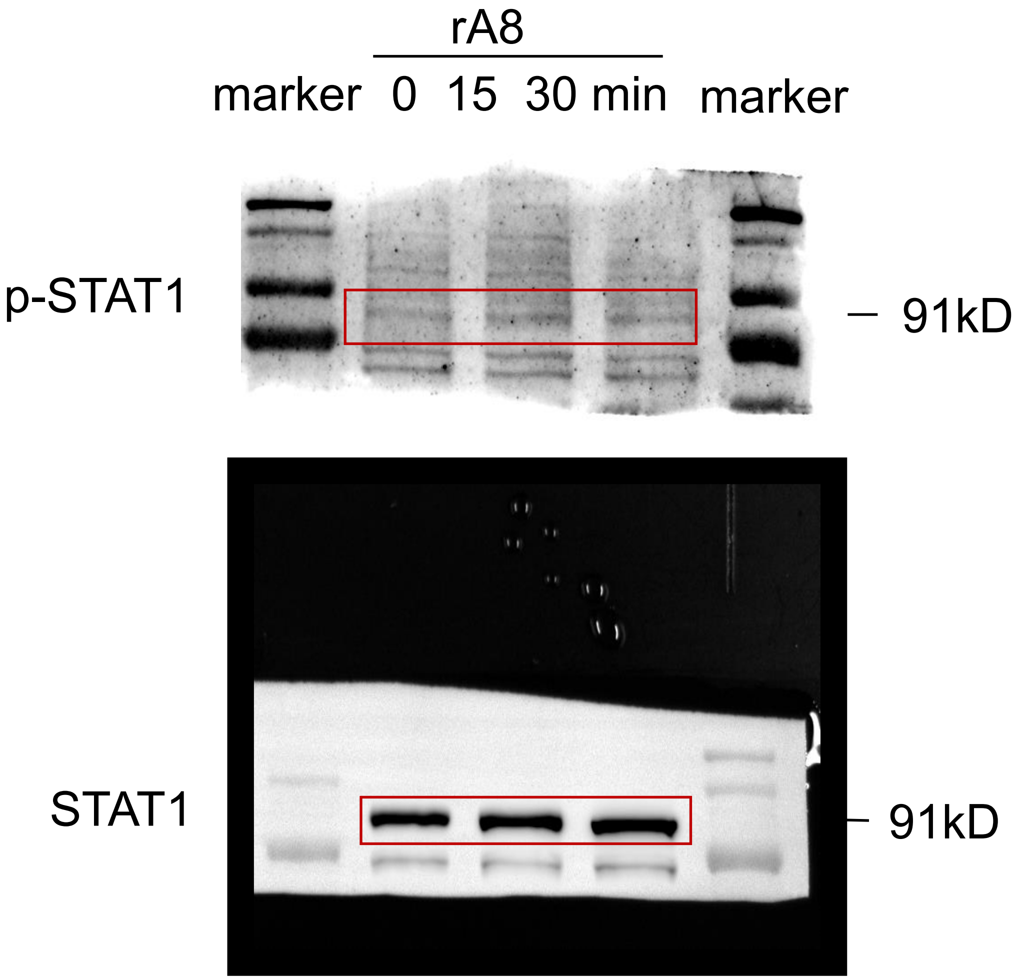

The red box indicates the region used in the final figure. Membranes were probed sequentially for target proteins and loading controls as indicated. Molecular weight markers are shown for reference.

Fig. S4F P38 full blot

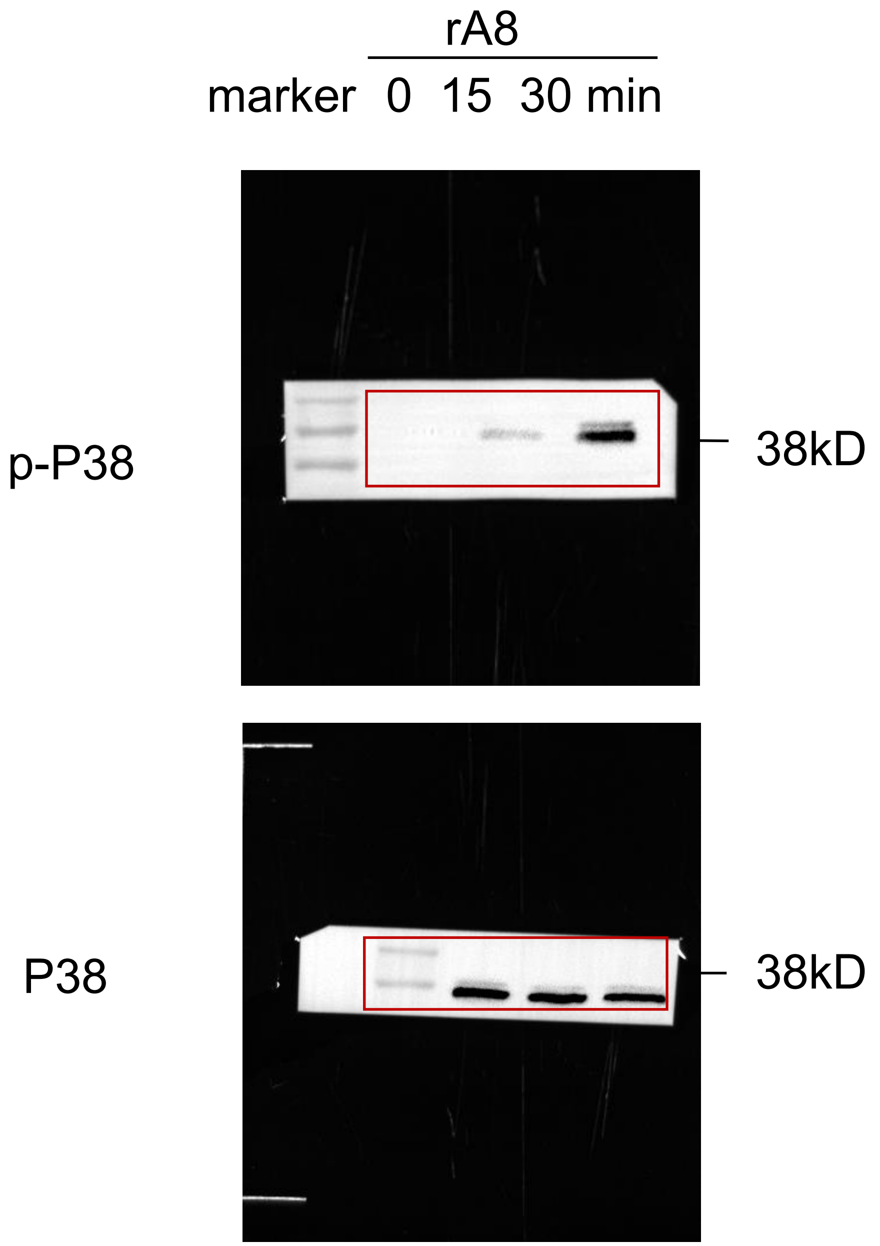

The red box indicates the region used in the final figure. Membranes were probed sequentially for target proteins and loading controls as indicated. Molecular weight markers are shown for reference.
